# Rationally Repurposing Ruxolitinib (*Jakafi*^®^) as a Solid Tumor Therapeutic

**DOI:** 10.3389/fonc.2016.00142

**Published:** 2016-06-13

**Authors:** Mehrad Tavallai, Laurence Booth, Jane L. Roberts, Andrew Poklepovic, Paul Dent

**Affiliations:** ^1^Department of Biochemistry and Molecular Biology, Virginia Commonwealth University, Richmond, VA, USA; ^2^Department of Medicine, Virginia Commonwealth University, Richmond, VA, USA

**Keywords:** ruxolitinib, JAK1/2, afatinib, ERBB1, mitophagy, chaperone, AIF

## Abstract

We determined whether the approved myelofibrosis drug ruxolitinib (Jakafi^®^), an inhibitor of Janus kinases 1/2 (JAK1 and JAK2), could be repurposed as an anti-cancer agent for solid tumors. Ruxolitinib synergistically interacted with dual ERBB1/2/4 inhibitors to kill breast as well as lung, ovarian and brain cancer cells. Knock down of JAK1/2 or of ERBB1/2/3/4 recapitulated on-target drug effects. The combination of (ruxolitinib + ERBB1/2/4 inhibitor) rapidly inactivated AKT, mTORC1, mTORC2, STAT3, and STAT5, and activated eIF2α. In parallel, the drug combination reduced expression of MCL-1, BCL-XL, HSP90, HSP70, and GRP78, and increased expression of Beclin1. Activated forms of STAT3, AKT, or mTOR prevented the drug-induced decline in BCL-XL, MCL-1, HSP90, and HSP70 levels. Over-expression of chaperones maintained AKT/mTOR activity in the presence of drugs and protected tumor cells from the drug combination. Expression of dominant negative eIF2α S51A prevented the increase in Beclin1 expression and protected tumor cells from the drug combination. Loss of mTOR activity was associated with increased ATG13 S318 phosphorylation and with autophagosome formation. Autophagosomes initially co-localized with mitochondria and subsequently with lysosomes. Knock down of Beclin1 suppressed: drug-induced mitophagy; the activation of the toxic BH3 domain proteins BAX and BAK; and tumor cell killing. Knock down of apoptosis-inducing factor (AIF) protected tumor cells from the drug combination, whereas blockade of caspase 9 signaling did not. The drug combination released AIF into the cytosol and increased nuclear AIF: eIF3A co-localization. A 4-day transient exposure of orthotopic tumors to (ruxolitinib + afatinib) profoundly reduced mammary tumor growth over the following 35 days. Re-grown tumors exhibited high levels of BAD S112 phosphorylation and activation of ERK1/2 and NFκB. Our data demonstrate that mitophagy is an essential component of (ruxolitinib + ERBB inhibitor) lethality and that this drug combination should be explored in a phase I trial in solid tumor patients.

## Introduction

Immune cell activation in general and particularly during rheumatoid arthritis progression requires signaling by Janus kinases (JAK1, JAK2, JAK3). Thus pharmaceutical companies, attacking these kinases as drugable targets, have developed several FDA-approved agents in the hope of reducing the negative sequelae of arthritis and of semi-tumorigenic myelo-proliferative disorders: Jakafi and Xeljanz, respectively (1–4). Jakafi (ruxolitinib) inhibits JAK1 and JAK2, whereas Xeljanz (tofacitinib) inhibits JAK3 and to a lesser extent JAK1. In the field of cancer research and therapy ruxolitinib has been used, logically based on its immune cell actions, in the treatment of myelo-proliferative disorders, myelogenous neoplasms, and auto-immune diseases, such as psoriasis (5). The Janus kinases phosphorylate signal transducers and activators of transcription (STAT) transcription factors on tyrosine resulting in factor dimerization and nuclear localization, and eventually activation of various target genes (6–10). Thus, mutated active forms of Janus kinases or the actions of mutated activated growth factor receptors through autocrine loops cause constitutive activation of the STAT1/STAT3/STAT5 transcription factors that promote the malignant phenotype. Growth factor receptors, such as ERBB1, and c-MET also have been shown to phosphorylate STAT factors on tyrosine residues thereby promoting dimerization/activation (11, 12). Cyto-protective genes activated by STAT transcription factors are many and include those coding for anti-apoptotic genes, such as MCL-1, BCL-XL, BCL-2, survivin, HSP90, and HSP70; proliferation regulatory genes, such as Cyclin D1, Cyclin B, c-Jun, and c-Fos; and angiogenesis promoting genes, such as HIF1α, and growth factors, such as IL-6, FGF, EGF, and VEGF (13–20).

It is well known that in the majority of tumor cell isolates across all malignancies, i.e., cells that are generally not addicted to any one specific single driving oncogene, that in order to kill the tumor cell effectively *in vitro* and *in vivo* requires the combinatorial use of two or more modulators of signal transduction pathways. For example, published studies from this laboratory combining (MEK1/2 inhibitors + CHK1 inhibitors); (sorafenib/regorafenib + PI3K/AKT inhibitors); (MMF and XRT/Temozolomide); and (HSP90 inhibitors + MEK1/2 inhibitors) are good illustrations of this dual pathway inhibition to kill concept (21–29).

More recent studies from this laboratory have extended the dual pathway inhibition killing concept by the use of multiplex antibody array assays on drug-treated tumors that permit simultaneous analyses of plasma cytokine levels and the activity status of multiple signal transduction parameters in tumors/tumor cells surviving the dual pathway inhibition treatment. For example, in 2015, we published that the drugs sorafenib/regorafenib interacted with phosphodiesterase 5 inhibitors, such as sildenafil (Viagra) and tadalafil (Cialis) in a synergistic fashion to kill tumor cells *in vitro* and *in vivo* (28). Based on multiplex assays of plasma and tumor material from the rodent tumor studies contained within this paper, we discovered that these drug combination treatments caused a compensatory activation of ERBB1/2/4-PI3K-AKT in the liver and colorectal tumor cells surviving the (sorafenib/regorafenib + sildenafil) drug treatments. *Nota bene*: tumors from two different GI tissues exhibited very similar compensatory/evolutionary survival signaling responses. And, the combination of an ERBB1/2/4 inhibitor or a PI3K inhibitor or an AKT inhibitor with (sorafenib/regorafenib + sildenafil) was then shown to profoundly enhanced tumor cell killing *in vitro*. New phase I and phase II trials have opened at VCU combining (regorafenib + sildenafil) and (sorafenib + sildenafil + valproate) for all solid tumor patients and for glioblastoma (GBM) patients, respectively (NCT02466802; NCT02337426).

Very recently, we have shown that a major novel component of the biology of the drugs OSU-03012 (AR12), sorafenib and pazopanib, but to a much lesser extent regorafenib, afatinib or ruxolitinib, is the actual inhibition of protein chaperone ATPase activities and, hence, reducing the ability of chaperones to interact with each other and with their many client proteins (30–33). The present studies were performed to determine whether the myeloid-proliferative disorder drug ruxolitinib could be repurposed for use as an anti-cancer therapy for solid tumors. The plasma C max for ruxolitinib in patients undergoing standard of care therapy is ~136 μM with >95% of the drug protein bound, although significantly higher doses of the drug were safely administered to patients during drug development clinical evaluations. Thus, *in vitro* studies in the present manuscript generally use ruxolitinib-phosphate at a concentration of 2.5 μM or less to reflect the probable safe achievable level of bioactive drug in a patient.

## Materials and Methods

### Materials

Lapatinib tosylate, Afatinib, Neratinib, Vandetanib, and Ruxolitinib-phosphate were purchased from Selleckchem (Houston, TX, USA). Trypsin–EDTA, DMEM, RPMI, penicillin-streptomycin were purchased from GIBCOBRL (GIBCOBRL Life Technologies, Grand Island, NY, USA). Mono-methyl fumarate was from Sigma (St. Louis, MO, USA). Cells were purchased from the ATCC and were not further validated beyond that claimed by ATCC. Cells were re-purchased every ~6 months. Primary human GBM cells, developed by Dr. C.D. James when at the Mayo Clinic (Rochester, MN, USA) have been described previously. ADOR non-small cell lung cancer cells are personal a donation from the patient to the Dent laboratory. *De novo* cisplatin resistant “Spiky” ovarian cancer cells, a patient-derived explant (PDX) model, were kindly provided by Dr. Karen Paz (Champions Oncology, NJ, USA). The plasmid to express GRP78 was kindly provided to the Dent laboratory by Dr. A.S. Lee (University of Southern California Los Angeles, CA, USA). The plasmids to express HSP27, eIF2α S51A, kinase-inactive PERK, and all others listed in this manuscript were purchased from Addgene (Cambridge, MA, USA). Commercially available validated short hairpin RNA molecules to knock down RNA/protein levels were from Qiagen (Valencia, CA, USA) or were supplied by collaborators. Reagents and performance of experimental procedures were described in Refs. (30–33).

### Methods

#### Culture and *In Vitro* Exposure of Cells to Drugs

All cell lines were cultured at 37°C [5% (v/v CO_2_)] *in vitro* using RPMI supplemented with dialyzed 5% (v/v) fetal calf serum and 10% (v/v) Non-essential amino acids. *In vitro* drug treatments were from 100 mM stock solutions of each drug and the maximal concentration of Vehicle (DMSO) in media was 0.02% (v/v). Cells were not cultured in reduced serum media during any study in this manuscript.

#### Transfection of Cells with siRNA or with Plasmids

##### For Plasmids

Cells were plated and 24 h after plating, transfected. Plasmids expressing a specific mRNA (or siRNA) or appropriate vector control plasmid DNA was diluted in 50 μl serum-free and antibiotic-free medium (one portion for each sample). Concurrently, 2 μl Lipofectamine 2000 (Invitrogen) was diluted into 50 μl of serum-free and antibiotic-free medium (one portion for each sample). Diluted DNA was added to the diluted Lipofectamine 2000 for each sample and incubated at room temperature for 30 min. This mixture was added to each well/dish of cells containing 200 μl serum-free and antibiotic-free medium for a total volume of 300 μl, and the cells were incubated for 4 h at 37°C. An equal volume of 2× medium was then added to each well. Cells were incubated for 24 h, then treated with drugs.

##### Transfection for siRNA

Cells from a fresh culture growing in log phase as described above, and 24 h after plating transfected. Prior to transfection, the medium was aspirated and serum-free medium was added to each plate. For transfection, 10 nM of the annealed siRNA, the positive sense control double-stranded siRNA targeting GAPDH or the negative control (a “scrambled” sequence with no significant homology to any known gene sequences from mouse, rat or human cell lines) were used. Ten nanomolar siRNA (scrambled or experimental) was diluted in serum-free media. Four-microliter Hiperfect (Qiagen) was added to this mixture and the solution was mixed by pipetting up and down several times. This solution was incubated at room temp for 10 min, then added drop-wise to each dish. The medium in each dish was swirled gently to mix, then incubated at 37°C for 2 h. Serum-containing medium was added to each plate, and cells were incubated at 37°C for 24 h before then treated with drugs (0–24 h). Additional immuno-fluorescence/live–dead analyses were performed at the indicated time points.

#### Multiplex Assays

A MAGPIX multiplex instrument with associated software was purchased from Bio-Rad. The following Bio-Plex assay plates were used in our assays of mouse plasma for human cytokines: Bio-Plex Pro Human Cytokine Group I 4-plex (Y500023JM2); Human CYTO STD GRP II 23-PLEX (171D60001); Human CYTO HGF set (171B6008M); Human CYTO SDF-1a set (171B6019M); Pro Human Cancer 2 18-plex (171AC600M); BP Pro TGF-B 3-PLEX (171W4001M). Mouse plasma was assayed according to the instructions provided by Bio-Rad to assess human cytokine levels derived from the BT474 tumors. The following Bio-Plex assay plates were used in our assays for tumor cell signal transduction proteins: Bio-Plex Bio-Plex Pro Phospho-protein magnetic 8-plex Assay (LQ00004IXUYDC4); Bio-Plex Pro Phospho-protein magnetic 15-plex Assay (LQ000064Q3MJ1). Tumor lysates were assayed according to the instructions provided by Bio-Rad to assess human signaling changes derived from the BT474 tumors.

#### Detection of Cell Viability, Protein Expression, and Protein Phosphorylation by Immuno-Fluorescence Using a Hermes WiScan Machine

The vast majority of the *in vitro* analyses in these manuscripts, and the present set of studies, used a novel approach to detect gross changes in protein expression and protein phosphorylation: a Hermes WiScan wide field microscope (http://www.idea-bio.com/). Set at 10× magnification, in a 96-well plate format, gross assessments of protein expression/phosphorylation can be made, i.e., an “in-cell western,” using unbiased pre-programed electronic data acquisition, much as has previously been performed for the last 45 years using SDS-PAGE and western immunoblotting. However, the Hermes system permits 96 samples to be measured simultaneously in up to three fluorescent color channels in contrast to our Odyssey infra-red machine for western blotting that has two channels and is limited to ~20 samples per gel. Using the Hermes system has an additional benefit: while western immunoblotting often “loses” proteins that denature and form detergent insoluble complexes that do not electrophorese down the gel, using the Hermes system all cellular proteins have the potential of being detected due to its *in situ* non-denatured methodology (30–33). The machine also has in-built software to assess changes in fluorescent intensity thereby making the quantitation of signal intensities straight-forward. Cells (4 × 10^3^) are plated into each well of a 96-well plate, and cells permitted to attach and grow for the next 18 h. Based on the experiment, after 18 h, cells are then either genetically manipulated, or are treated with drugs. For genetic manipulation, cells are transfected with plasmids or siRNA molecules and incubated for an additional 24 h. Cells are treated with vehicle control or with drugs at the indicated final concentrations, alone or in combination. Cells are then isolated for processing at various times following drug exposure. The 96-well plate is centrifuged/cyto-spun to associate dead cells (for live–dead assays) with the base of each well. For live–dead assays, after centrifugation, the media is removed and cells treated with live–dead reagent (Thermo Fisher Scientific, Waltham, MA, USA) and, after 10 min, this is removed and the cells in each well are visualized in the Hermes instrument at 10× magnification. Green cells = viable; yellow/red cells = dying/dead. The numbers of viable and dead cells were counted manually from three images taken from each well combined with data from another two wells of separately treated cells (i.e., the data are the mean cell dead from nine data points from three separate exposures). For immuno-fluorescence studies, after centrifugation, the media is removed and cells are fixed in place and permeabilized using ice cold PBS containing 0.4% paraformaldehyde and 0.5% Triton X-100. After 30 min, the cells are washed three times with ice cold PBS and cells are pre-blocked with rat serum for 3 h. Cells are then incubated with a primary antibody to detect the expression/phosphorylation of a protein (usually at 1:100 dilution from a commercial vendor) overnight at 37°C. Cells are washed three times with PBS followed by application of the secondary antibody containing an associated fluorescent red or green chemical tag. After 3 h of incubation, the antibody is removed and the cells washed again. The cells are visualized at either 10× or 60× in the Hermes machine for imaging assessments. All immunofluorescent images for each individual protein/phospho-protein are taken using the identical machine settings so that the levels of signal in each image can be directly compared to the level of signal in the cells treated with drugs. Similarly, for presentation, the enhancement of image brightness/contrast using PhotoShop CS6 is simultaneously performed for each individual set of protein/phospho-protein to permit direct comparison of the image intensity between treatments.

For SDS-PAGE and immunoblotting, cells were plated at 5 × 10^5^ cells/cm^2^ and treated with drugs at the indicated concentrations and after the indicated time of treatment, lysed in whole-cell lysis buffer (0.5 M Tris-HCl, pH 6.8, 2% SDS, 10% glycerol, 1% β-mercaptoethanol, 0.02% bromophenol blue), and the samples were boiled for 30 min. The boiled samples were loaded onto 10–14% SDS-PAGE and electrophoresis was run overnight (10–100 μg/lane based on the gel size). Proteins were electrophoretically transferred onto 0.22-μm nitrocellulose, and immunoblotted with various primary antibodies against different proteins. Antibodies used include: HSP90 (E289) (Cell Signaling); HSP90 (#2928) (Abcam); HSP90 (ab195575) Abcam; HSP90 3G3 (13495) (Abcam); GRP78 (50b12) (31772) (Cell Signaling); GRP78 (ab191023) Abcam; GRP78 (ab103336) Abcam; GRP78 (N-20) (sc-1050) Santa Cruz; HSP27 (G31) (2402P) Cell Signaling); HSP27 (EP1724Y) (ab62339) Abcam; HSP27 (H-77) (sc-9012) Santa Cruz; HSP27 (LS-C31836) Lifespan science Corp. Other antibodies were as used in prior studies by the laboratory.

#### Animal Studies

Animal studies were performed according to Federal Law and under an approved Virginia Commonwealth University IACUC protocol (#AD10001065). Athymic nude mice (~20 g) were injected with 1 × 10^7^ BT474 cells into their fourth mammary fat pad (10 animals per treatment group; 4 groups; a total of 40 mice ± SEM). Tumors were permitted to form for 7 days with tumors at that time exhibiting a mean volume of ~15 mm^3^. This is the equivalent of a 3.7 cm^3^ massed tumor in the breast of a 50 kg adult female patient. Athymic mice were treated by oral gavage once every day for 4 days as indicated in the Figure and Figure Legend with vehicle (Cremophore); with ruxolitinib (50 mg/kg BID) on days 1–4; with MMF on days 1–4 (50 mg/kg); and with afatinib (25 mg/kg QD) on days 1–4. After cessation of drug treatment, tumors are again callipered, and tumor volume was assessed up to 35 days later.

#### Data Analysis

Comparison of the effects of various treatments was performed using one way analysis of variance and a two-tailed Student’s *t*-test. Statistical examination of *in vivo* animal survival data utilized log-rank statistical analyses between the different treatment groups. Differences with a *p*-value of <0.05 were considered statistically significant. Experiments shown are the means of multiple individual points from multiple experiments (±SEM).

## Results

**Figure 1:**
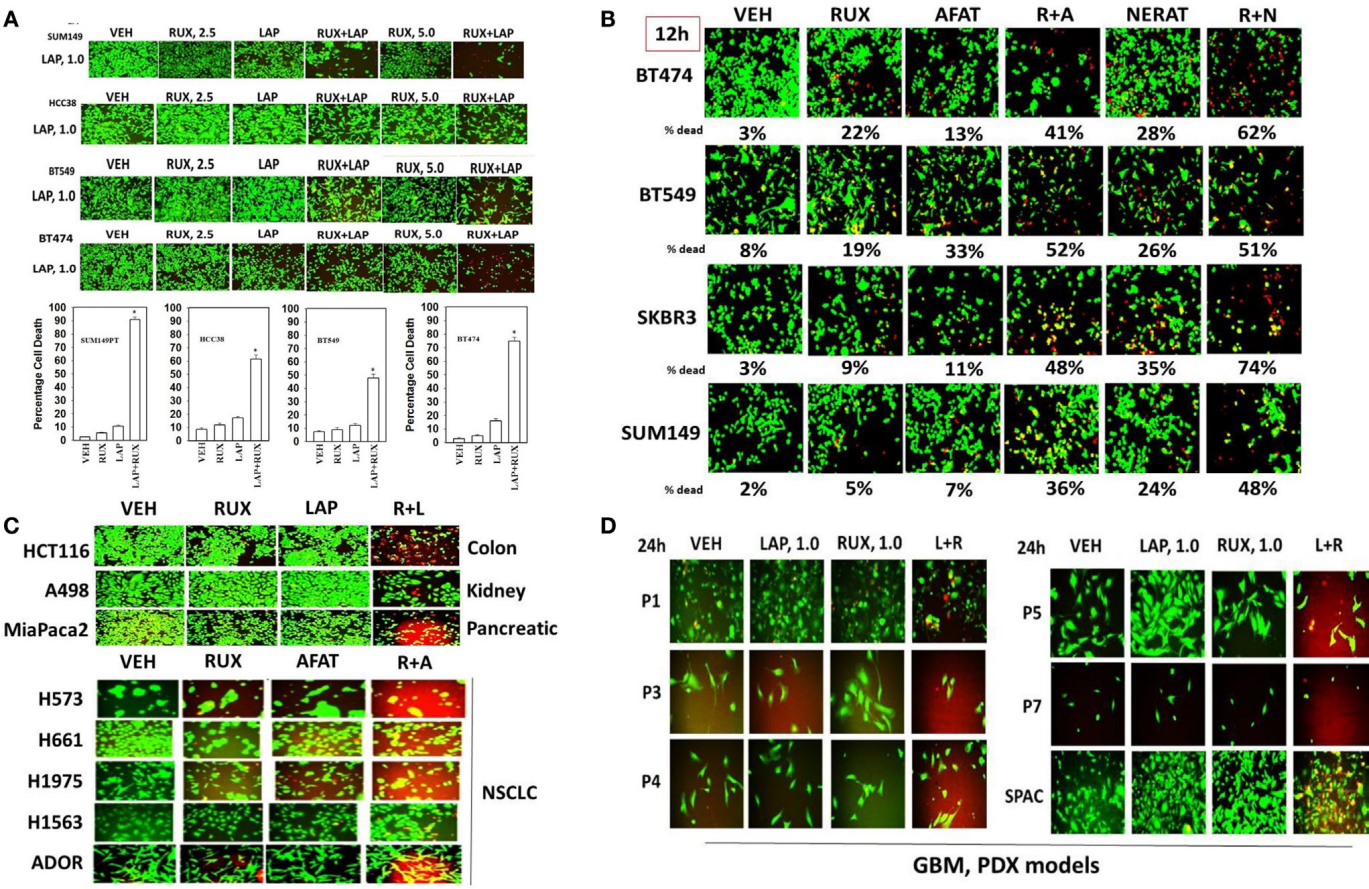
**The JAK1/2 inhibitor Ruxolitinib interacts with the ERBB1/2/4 inhibitor Lapatinib to kill breast cancer cell lines that: lack PTEN; over-express ERBB2; and are classified as triple negative**. **(A)** SUM149, BT549, HCC38, and BT474 cells were treated with vehicle control, ruxolitinib-phosphate (2.5/5.0 μM), lapatinib tosylate (1.0 μM) or the drugs in combination. Twenty-four hours later, cell viability was assessed using a live/dead assay in a Hermes WiScan microscope at 10× magnification (*n* = 3 ± SEM) *p < 0.05 greater than vehicle control. **(B)** BT474, BT549, SKBR3, and SUM149 cells were treated with vehicle control, ruxolitinib-phosphate (2.5 μM), afatinib (1.0 μM), neratinib (1.0 μM), or the drugs in combination as indicated. Twenty-four hours later, cell viability was assessed using a live/dead assay in a Hermes WiScan microscope at 10× magnification (*n* = 3 ± SEM). **(C)** HCT116 (colon); A498 (renal); Mia Paca2 (pancreatic) and multiple NSCLC lines including the July 2015 PDX isolate ADOR; cancer cells were treated with vehicle control, ruxolitinib-phosphate (2.5 μM), lapatinib (2.0 μM) or the drugs in combination as indicated. **(D)** Primary human glioblastoma cells (P1-P7) and a PDX model of recurrent medulloblastoma (SPAC) cancer cells were treated with vehicle control, ruxolitinib-phosphate (1.0 μM), lapatinib (1.0 μM) or the drugs in combination as indicated. Twenty-four hours later, cell viability under all conditions was assessed using a live/dead assay in a Hermes WiScan microscope at 10× magnification.

Our earliest studies determined whether the ERBB1/2/4 inhibitor lapatinib interacted with the JAK1/2 inhibitor ruxolitinib to kill breast cancer cells. In a dose-dependent fashion, ruxolitinib interacted with lapatinib to kill within 24 h: SUM149; BT549; HCC38; and BT474 mammary tumor cells (Figure 1A). Of note, SUM149, BT549, and HCC38 cells are considered to be “triple negative” and do not over-express ERBB2; BT549 and HCC38 cells lack PTEN function. Similar tumor cell killing data to that using lapatinib as shown in Figure 1 were obtained using the second- and third-generation ERBB1/2/4 inhibitors afatinib, neratinib, sapatinib, tagrisso, and poziotinib (Figure 1B, not shown). Of note, the more recently developed second/third-generation suicide ERBB receptor inhibitors were more capable of rapidly interacting with ruxolitinib and at lower concentrations, than was the first-generation inhibitor lapatinib, in terms of tumor cell killing. The lethal interaction between ERBB1/2/4 inhibitors and ruxolitinib was not only discovered in mammary tumor cells but was also found in HCT116 colon, A498 renal and MiaPaca2 pancreatic and multiple NSCLC cells, including the July 2015 ERBB3-dependent ADOR isolate (Figure 1C). In multiple fresh low passage 2012–2015 PDX isolates of human GBM and medulloblastoma (SPAC), lapatinib and ruxolitinib interacted to reduce growth and kill tumor cells (Figure 1D). Collectively, these data also imply that tumor cells expressing activated RAS proteins or lacking the tumor suppressor PTEN are killed by the drug combination.

**Figure 2:**
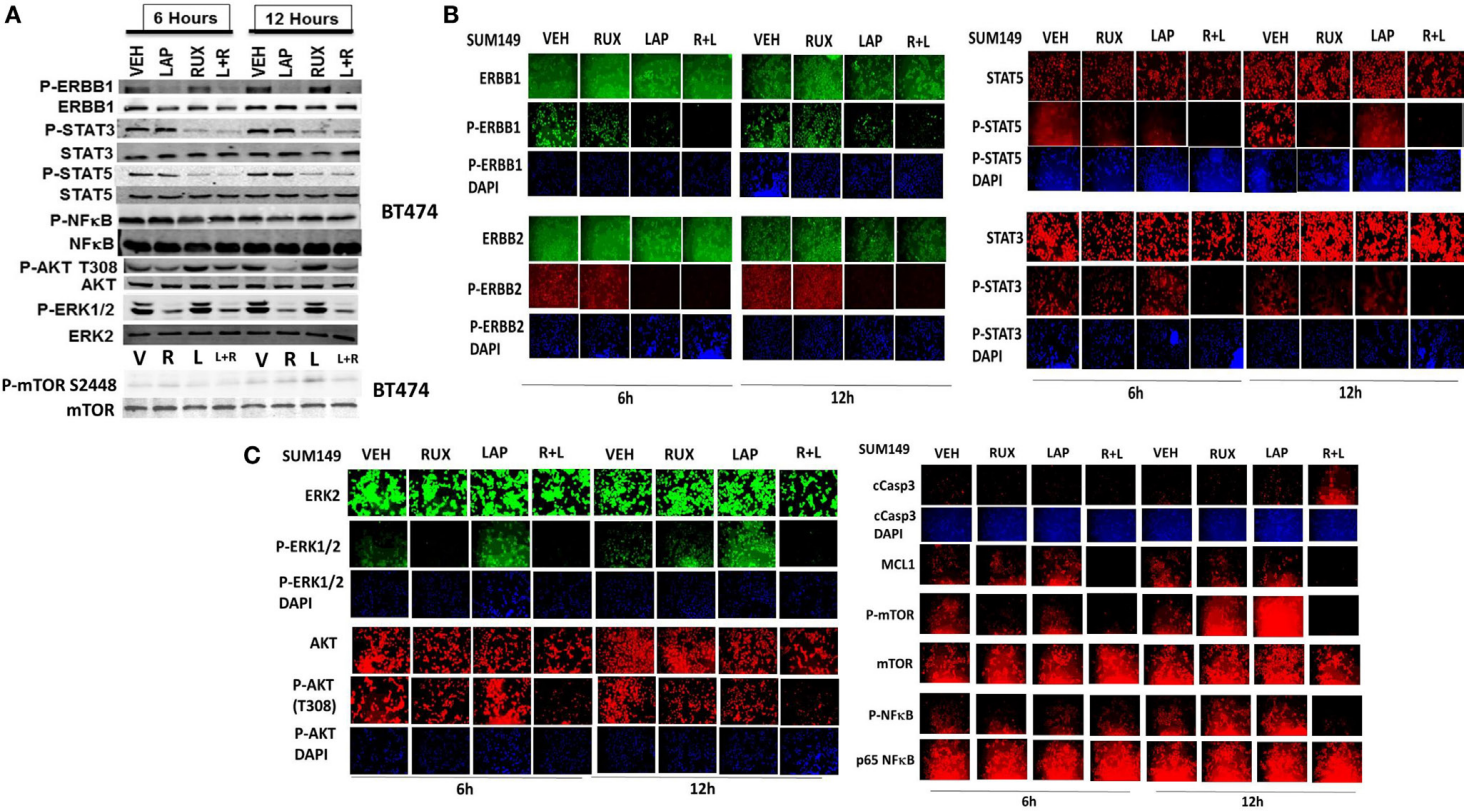
**Lapatinib and ruxolitinib interact to inactivate AKT, mTOR, STAT3, STAT5, and ERK1/2 as judged by western blotting**. **(A)** BT474 cells were treated with vehicle control, ruxolitinib (1.0 μM), lapatinib (1.0 μM) or the drugs in combination for 6 h and for 12 h. At each time point cells were lysed with RIPA buffer and clarified by centrifugation. Ten milligram of protein from each lysate was subjected to SDS-PAGE on 10% gels. Proteins were transferred to 0.2 μm nitrocellulose and probed with antibodies generated against the indicated proteins and phospho-proteins. Bands were imaged using a first generation Odyssey Infra-Red imager at 300 dpi. (*n* = 3 ± SEM). **(B,C)** SUM149 cells were treated with vehicle control, ruxolitinib (1.0 μM), lapatinib (1.0 μM), or the drugs in combination for 6 h and for 12 h. At each time point, cells were fixed in place and permeabilized using 0.5% Triton X100. Immuno-fluorescence was performed on native proteins to detect the total expression and phosphorylation levels of ERBB1, ERBB2, STAT3, STAT5, ERK1/2, AKT T308, mTOR S2448; NFκB p65 S536, MCL-1, and cleaved caspase 3 (cCasp3) (*n* = 3 ± SEM).

**Figure 3:**
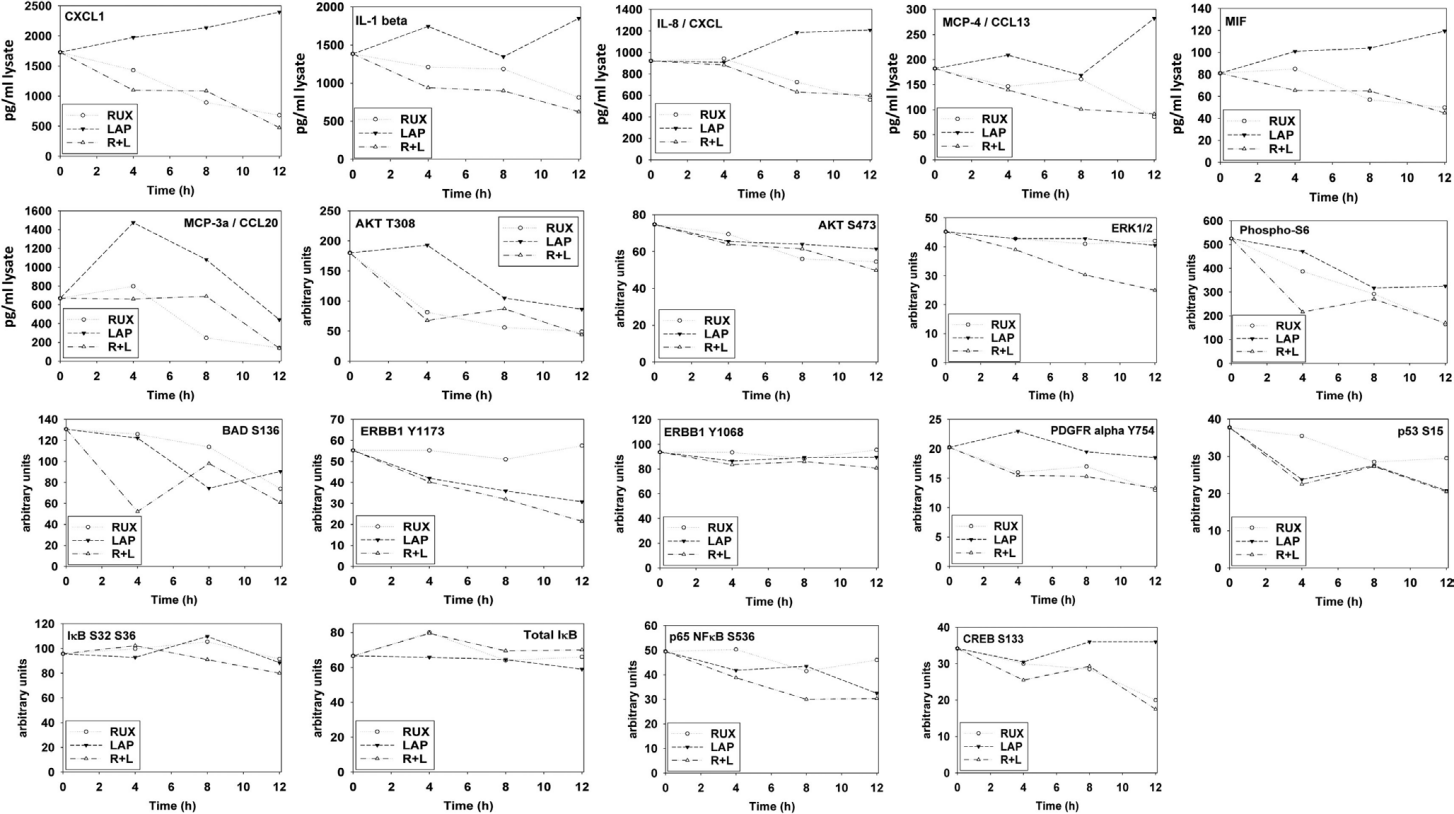
**Multiplex antibody array assays of SUM149 cells treated with ruxolitinib, lapatinib, or the drug combination**. SUM149 cells were treated with vehicle control, ruxolitinib (1.0 μM), lapatinib (1.0 μM), or the drugs in combination for 4 h, for 8 h, and for 12 h. At each time point, cells were lysed and clarified by centrifugation. Clarified tumor cell lysates were then subjected to multiplex assays as described in the section “Methods” to detect the tumor lysate levels of the indicated cytokines and phosphorylation status of signal transduction proteins in the tumor using a Bio-Rad MAGPIX multiplex instrument (*n* = 3 ± SEM).

Ruxolitinib and ERBB receptor inhibitors are known to modulate the functions and activities of many intracellular signal transduction pathways, and we next explored the impact our ruxolitinib based drug combination had on cell signaling processes. In BT474 cells as judged by western immunoblotting lapatinib and ruxolitinib in combination caused prolonged inhibition of the phosphorylation of ERBB1, ERBB2, ERK1/2, AKT, mTOR, STAT3, STAT5, and the phosphorylation of p65 NFκB (Figure 2A; effects all >50%, *p* < 0.05). Very similar data were obtained in triple-negative SUM149 cells when the changes in cell signaling parameters were measured using *in situ* immuno-fluorescence on native proteins in a Hermes WiScan machine at 10× magnification (Figures 2B,C). Assessments of changes in cell signaling using multiplex antibody array analyses in a Bio-Rad MAGPIX machine were also very similar to data generated using western blotting and immuno-fluorescence (Figure 3). The drug combination, as assessed by immuno-fluorescence, by 12 h had reduced the phosphorylation of ERK1/2, AKT T308, STAT3, and STAT5 by >75% (*p* < 0.05 less than vehicle control treated). From our multiplex antibody array analyses on *in vitro* cell lysates, we discovered that the combination of (ruxolitinib + lapatinib) did not alter IκB expression or its S32 S36 phosphorylation but did reduce NFκB p65 S536 phosphorylation, and decreased the expression of multiple protective and pro-inflammatory cytokines whose expression is regulated by NFκB, including CXCL-1, IL-1 beta, IL-8, CCL13, MIF, and CCL20. As assessed by both western immunoblotting and by immuno-fluorescence, 12 h after exposure, lapatinib as a single agent had enhanced mTOR activity that is presumably a form of compensatory survival mechanism, an effect that was blocked by co-exposure with ruxolitinib.

**Figure 4:**
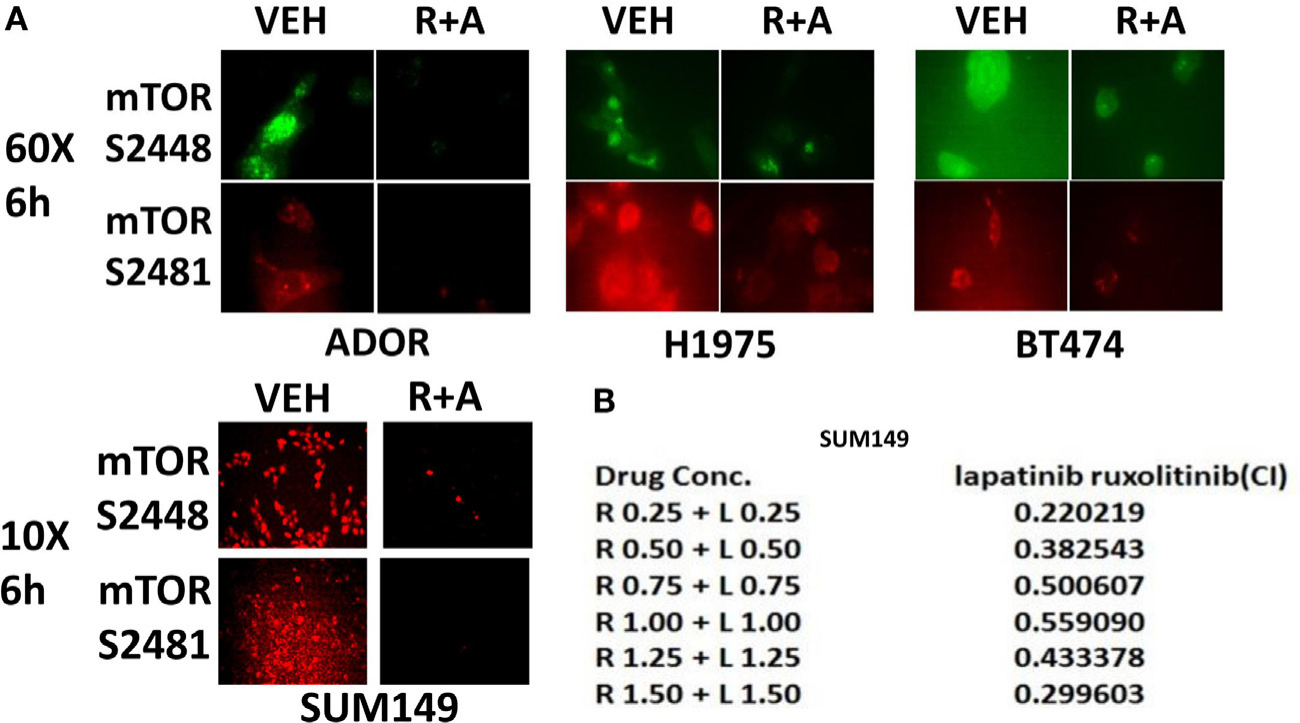
**The JAK1/2 inhibitor Ruxolitinib interacts with the ERBB1/2 inhibitor afatinib to reduce the phosphorylation of mTOR at Serine 2448 and Serine 2481: inactivation of mTORC1 and mTORC2**. **(A)** BT474, ADOR, SUM149, and H1975 cells were treated with vehicle control or [ruxolitinib (2 μM) + afatinib (2 μM)] for 6 h. After 6 h, cells were fixed in place and immuno-fluorescence was performed to detect at 60× magnification or at 10× as indicated, the phosphorylation status of mTOR S2448 and mTOR S2481. **(B)** Triple-negative SUM149 cells were plated (250–1,000) cells per well of a six well plate and 12 h after plating were treated with vehicle, ruxolitinib (0.25–1.5 μM; 0.5–2.5 μM), lapatinib (0.25–1.5 μM), or in combination at a constant ratio for 24 h, as indicated. The media was removed, cells were washed with drug-free media, and the cells cultured for another 10 days in drug-free media. Cell colonies were fixed, stained and groups of cells >50 were counted as colonies. The combination index (CI) for synergy was calculated using the Calcusyn for Windows program using the Cho and Tallalay Method (*n* = 2; 12 individual wells per data point ± SEM). A combination index of <0.70 indicates a strong level of tumor-killing synergy between the drugs.

The autophagy gate-keeper kinase mTOR occurs in two protein complexes termed mTORC1 and mTORC2. Phosphorylation of mTOR at Serine 2448 is a biomarker for mTORC1 activity, whereas phosphorylation of mTOR Serine 2481 is a biomarker for mTORC2 activity. We discovered that (ruxolitinib + afatinib) treatment reduced mTOR phosphorylation at both S2448 and S2481 in multiple breast and lung cancer cell lines, including the NSCLC PDX isolate ADOR, arguing that our drug combination was inactivating both the mTORC1 and mTORC2 signaling complexes (Figure 4A). And, in agreement with mTORC2 complex inactivation, the drug combination reduced AKT S473 phosphorylation (Figure 3). Finally, we determined whether transient exposure to lapatinib and ruxolitinib killed tumor cells in a synergistic fashion using median dose effect analyses on tumor cell colony formation. Ruxolitinib synergized with lapatinib to kill SUM149 mammary tumor cells in transient drug exposure colony formation assays, with combination index values significantly below 1.0 (Figure 4B).

**Figure 5:**
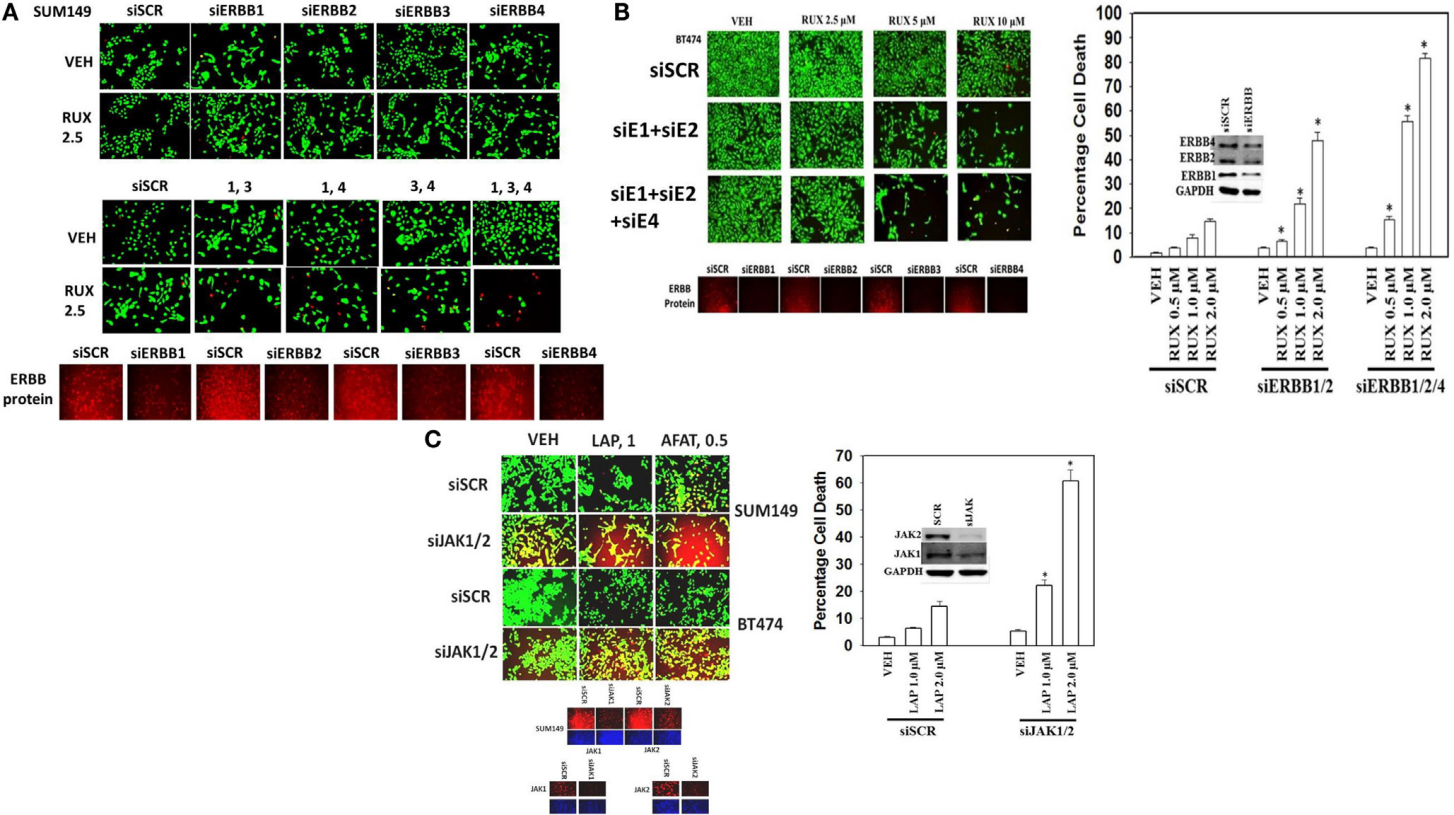
**Ruxolitinib synergizes with knock down of ERBB1/ERBB3/ERBB4 to kill SUM149 triple-negative breast cancer cells**. **(A)** SUM149 cells were transfected with a scrambled siRNA (siSCR) or with siRNA molecules to knock down the expression of ERBB1, ERBB2, ERBB3, ERBB4, either alone, or in combinations, as indicated in the figure. Twenty-four hours after transfection, cells were treated with vehicle control or ruxolitinib (2.5–10.0 μM), as indicated. Twenty-four hours later, cell viability was assessed using a live/dead assay in a Hermes WiScan microscope at 10× magnification (*n* = 3 ± SEM) *p < 0.05 greater than vehicle control. **(B)** BT474 cells were transfected with a scrambled siRNA (siSCR) or with siRNA molecules to knock down the expression of ERBB1, ERBB2, ERBB3, ERBB4, either alone, or in combinations, as indicated in the figure. Twenty-four hours after transfection, cells were treated with vehicle control or ruxolitinib (2.5–10.0 μM), as indicated. Twenty-four hours later, cell viability was assessed using a live/dead assay in a Hermes WiScan microscope at 10× magnification (*n* = 3 ± SEM) *p < 0.05 greater than vehicle control. **(C)** SUM149 and BT474 cells were transfected with a scrambled siRNA or with siRNA molecules to knock down expression of JAK1 and JAK2. Twenty-four hours after transfection, cells were treated with vehicle control, lapatinib (1 μM), or afatinib (0.5 μM). Twenty-four hours, later cell viability was assessed using a live/dead assay in a Hermes WiScan microscope at 10× magnification (*n* = 3 ± SEM) *p < 0.05 greater than vehicle control.

Next, using molecular tools, we defined which ERBB family members in different cell types were responsible for the interaction of ERBB receptor inhibitors and ruxolitinib. SUM149 cells were isolated from an inflammatory breast cancer patient whose tumor was defined as “triple negative” but which nevertheless in our hands expressed detectable basal levels of ERBB1, ERBB2, ERBB3, and ERBB4 by immuno-fluorescence of cells fixed *in situ* (Figure 5A). In SUM149 cells combined but not individual knock down of ERBB1, ERBB3, and ERBB4 strongly enhanced ruxolitinib toxicity. In BT474 cells, generally defined as a breast cancer cell type over-expressing ERBB2, we also detected expression of all four ERBB receptor members, and in this cell line combined knock down of ERBB1 and ERBB2 was sufficient to enhance ruxolitinib toxicity, though this effect was further enhanced by the additional knock down of ERBB4 (Figure 5B). GBM12 brain cancer cells, a PDX model isolated approximately a decade ago, express all ERBB receptors, including a full-length mutated active ERBB1, and, as we observed with the mammary SUM149 cells, the enhancement of ruxolitinib toxicity in GBM12 cells required combined knock down of ERBB1, ERBB3, and ERBB4 (data not shown). Ruxolitinib is claimed to be a specific inhibitor of the Janus kinases JAK1 and JAK2, but does not block JAK3 or TYK2, and we next determined whether knock down of JAK1 and JAK2 could account for the effects of ruxolitinib. Knock down of both JAK1 and JAK2 enhanced the lethality of lapatinib and of afatinib in the BT474 and SUM149 mammary carcinoma cells (Figure 5C).

**Figure 6:**
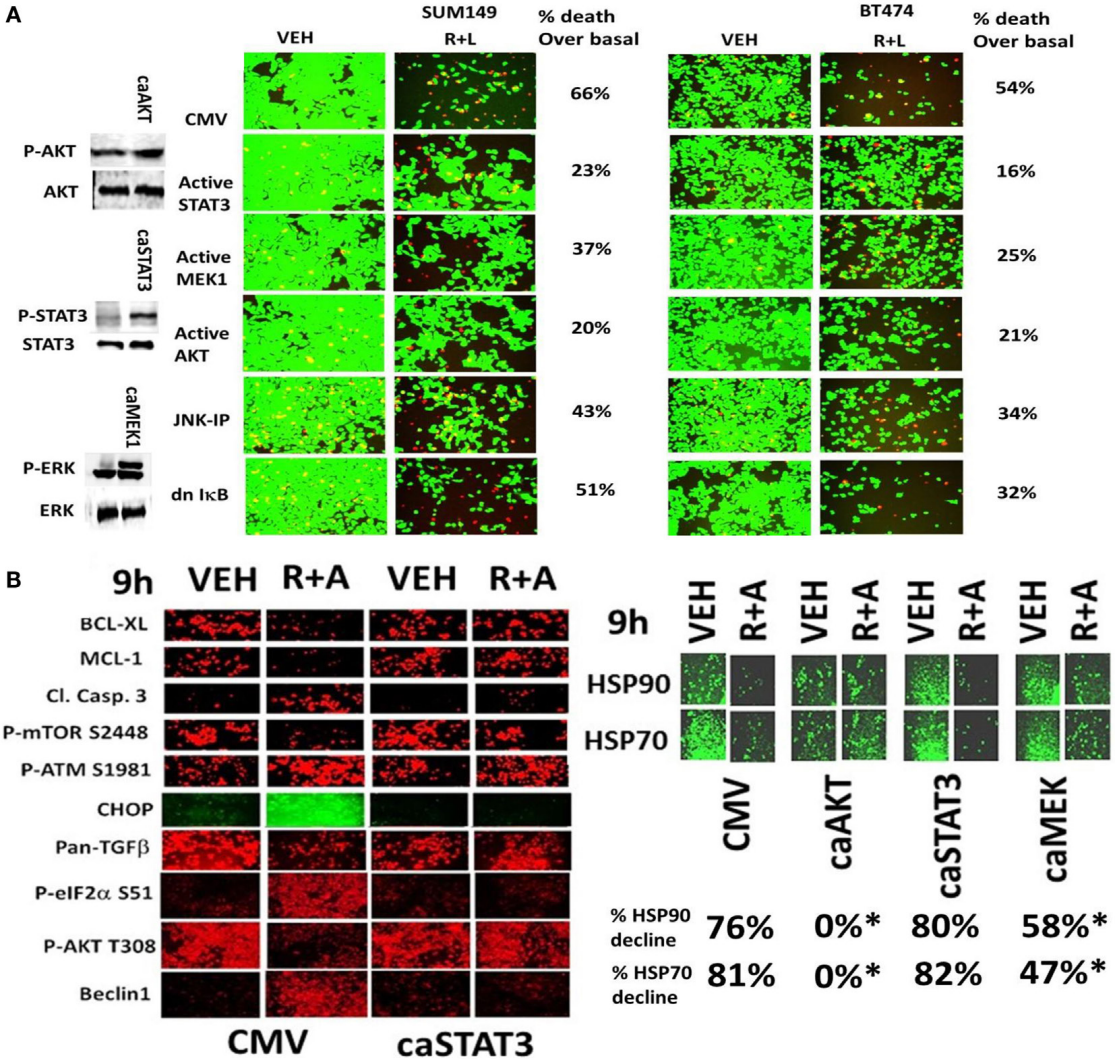
**Activated STAT3, activated AKT, or activated MEK1 protects mammary tumor cells from (ruxolitinib + lapatinib) treatment**. **(A)** SUM149 and BT474 cells were transfected with either an empty vector plasmid (CMV) or plasmids to express: activated STAT3; activated MEK1; activated AKT; or dominant negative IκB S32A S36A. In a portion of the CMV transfected cells, 15 min prior to drug treatment, cells were treated with the JNK inhibitory peptide (JNK-IP, 10 μM). Twenty-four hours after transfection, cells were treated with vehicle control; or ruxolitinib (1 μM) and lapatinib (1 μM) for 24 h. Twenty-four hours later, cell viability was assessed using a live/dead assay in a Hermes WiScan microscope at 10× magnification (*n* = 3 ± SEM). **(B)** SUM149 cells were transfected with empty vector plasmid (CMV) or a plasmid coding for activated STAT3 or a plasmid coding for activated AKT or a plasmid coding for activated MEK1, as indicated. Twenty-four hours after transfection, cells were treated with vehicle control or with [ruxolitinib (1.0 μM) + afatinib (1.0 μM)] for 6 h after which cells were fixed in place and permeabilized using 0.5% Triton X100. Immuno-fluorescence was performed at 10× magnification to detect the total expression and phosphorylation levels of the indicated proteins (*n* = 3 ± SEM) *p < 0.05 lower reduction than vehicle control.

**Figure 7:**
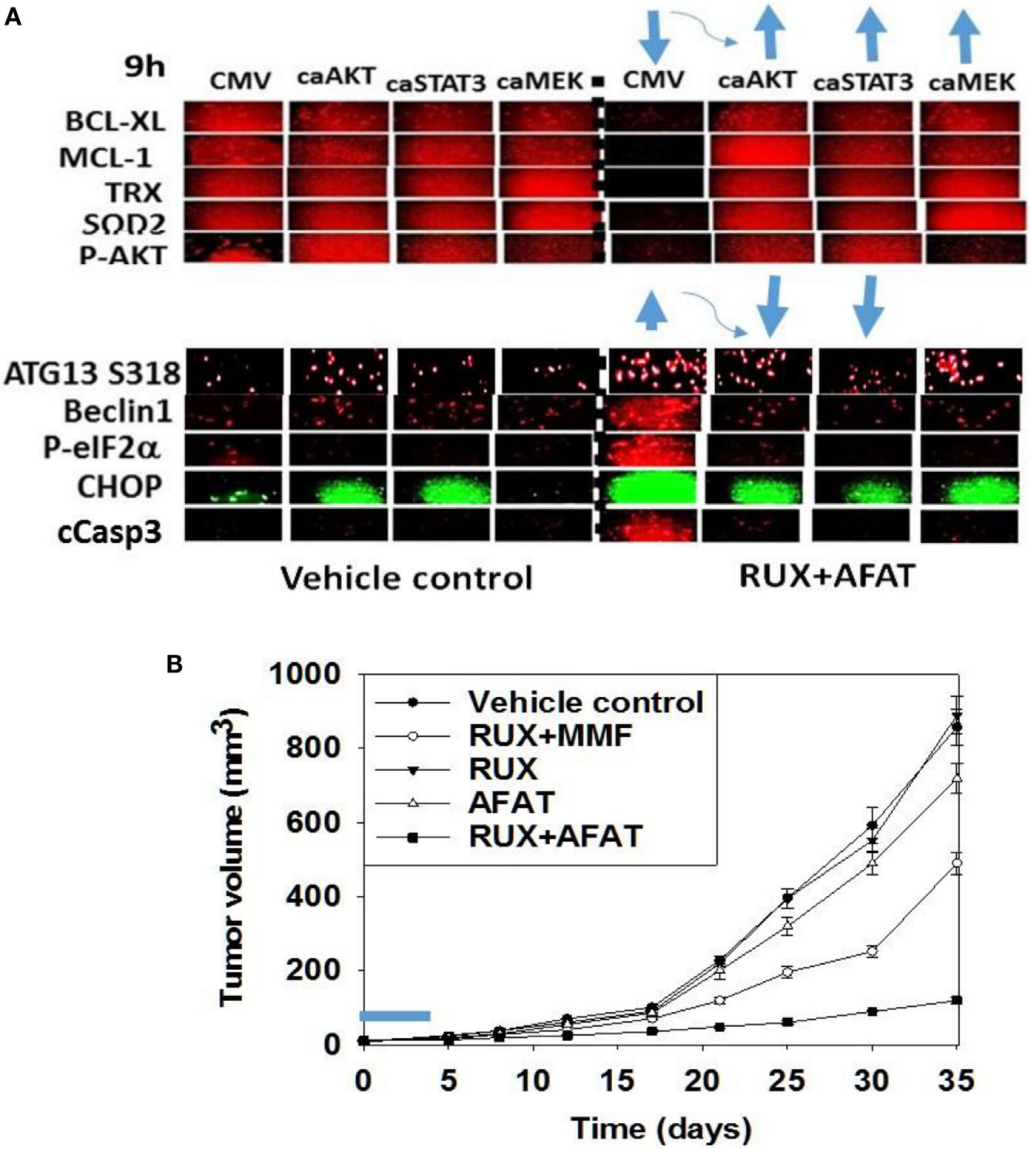
**Expression of activated STAT3 prevents the inactivation of AKT and mTOR, and the decline in BCL-XL and MCL-1 expression**. **(A)** SUM149 cells were transfected with empty vector plasmid (CMV) or a plasmid coding for activated STAT3 or a plasmid coding for activated AKT or a plasmid coding for activated MEK1, as indicated. Twenty-four hours after transfection, cells were treated with vehicle control or with [ruxolitinib (1.0 μM) + afatinib (1.0 μM)] for 6 h after which cells were fixed in place and permeabilized using 0.5% Triton X100. Immuno-fluorescence was performed at 10× magnification to detect the total expression and phosphorylation levels of the indicated proteins (*n* = 3 ± SEM). The blue arrows (upwards or downwards) represent statistically significant differences in fluorescence intensity. For vector control transfected cells treated with [Rux ± Afat] the comparison is to vector control transfected cells with vehicle treatment. For cells transfected with activated constructs and treated with [Rux ± Afat] the comparision is to vector control transfected cells treated with [Rux ± Afat] (all *p* < 0.05). **(B)** ruxolitinib and afatinib interact *in vivo* to reduce the growth of mammary carcinoma tumors. Athymic nude mice (~20 g) were injected with 1 × 10^7^ BT474 cells into their fourth mammary fat pad (10 animals per treatment group; four groups; a total of 40 mice ± SEM). Tumors were permitted to form for 7 days with tumors at that time exhibiting a mean volume of ~15 mm^3^. A 15 mm^3^ tumor in a 20 g mouse is the equivalent of a 3.7 cm^3^ tumor in a 50 kg patient. Athymic mice were treated by oral gavage once every day for 4 days: with vehicle (Cremophore); with MMF (50 mg/kg BID); with ruxolitinib (25 mg/kg BID) on days 1–4 and with afatinib (25 mg/kg QD) on days 1–4; and in the indicated combinations. After cessation of drug treatment tumors are again calipered and tumor volume was assessed up to 35 days later (±SEM).

In Figures 1–5, we demonstrated that the activities of ERK1/2, AKT, mTOR, STAT3, and NFκB p65 were being decreased for at least 12 h by our (ruxolitinib + ERBB inhibitor) drug combination and we next determined the impact of pathway inactivation(s) on tumor cell viability. In SUM149 triple-negative breast cancer cells, expression of an activated form of STAT3 or an activated form of AKT strongly inhibited the lethality of (ruxolitinib + lapatinib) treatment (Figure 6A). In BT474 cells expression of an activated form of STAT3, an activated form of MEK1 or an activated form of AKT also inhibited the lethality of (ruxolitinib + lapatinib) treatment. The (ruxolitinib + afatinib) drug combination rapidly reduced the expression of BCL-XL (by >70%, *p* < 0.05), MCL-1 (by >70%, *p* < 0.05), TGF-beta isoforms (by > 50%, *p* < 0.05) as well as the total protein levels of the chaperones HSP90 and HSP70 as measured using an antibody against the COOH-termini of the proteins; effects that were partially or fully reversed by expression of activated STAT3, activated AKT, or activated MEK1 (Figures 6B and 7A) (30). Of additional note, (ruxolitinib + afatinib) treatment also induced an endoplasmic reticulum stress response as judged by increased eIF2α S51 phosphorylation, and increased Beclin1 and CHOP expression. Expression of a dominant negative eIF2α S51A protein prevented the increases in Beclin1 and CHOP expression and partially protected tumor cells from (ruxolitinib + afatinib) exposure (data not shown). These findings are very similar to our recent clinically relevant data combining (pemetrexed + sorafenib) where the ability of this combination to cause an ER stress response and increase Beclin1 levels was key to tumor cell killing (32).

We then performed animal studies to define whether our *in vitro* drug combination studies translated *in vivo*. BT474 cells were implanted into the fourth mammary fat pad of athymic mice and tumors permitted to grow for 7 days, with initial starting tumor volume of ~15 mm^3^. N*ota bene*: an established 15 mm^3^ tumor volume in the fourth mammary fat pad of a 20 g mouse is the equivalent of a 3.7 cm^3^ tumor volume in the breast of a 50-kg female patient. Animals were treated for 4 days with vehicle control or with (ruxolitinib + afatinib) or (ruxolitinib + MMF) [see also (33)]. Treatment of animals with ruxolitinib alone or MMF alone did not alter tumor growth whatsoever, and treatment of animals with afatinib alone showed a trend for reduced BT474 tumor growth that was not statistically significant (Figure 7B, not shown). A 4-day treatment of mice with (ruxolitinib + afatinib) in combination significantly reduced the growth of BT474 tumors over a 35-day time course (Figure 7B, *p* < 0.001). Treatment of animals with (ruxolitinib + MMF) also significantly reduced tumor growth below control treated tumors, though to a lesser extent than (ruxolitinib + afatinib) (both, *p* < 0.05). Thus, the data in Figures 1–7 validate the combination of (ruxolitinib + ERBB1/2/4 inhibitor) and of (ruxolitinib + MMF) as putative anti-cancer therapies for mammary tumors (33).

**Figure 8:**
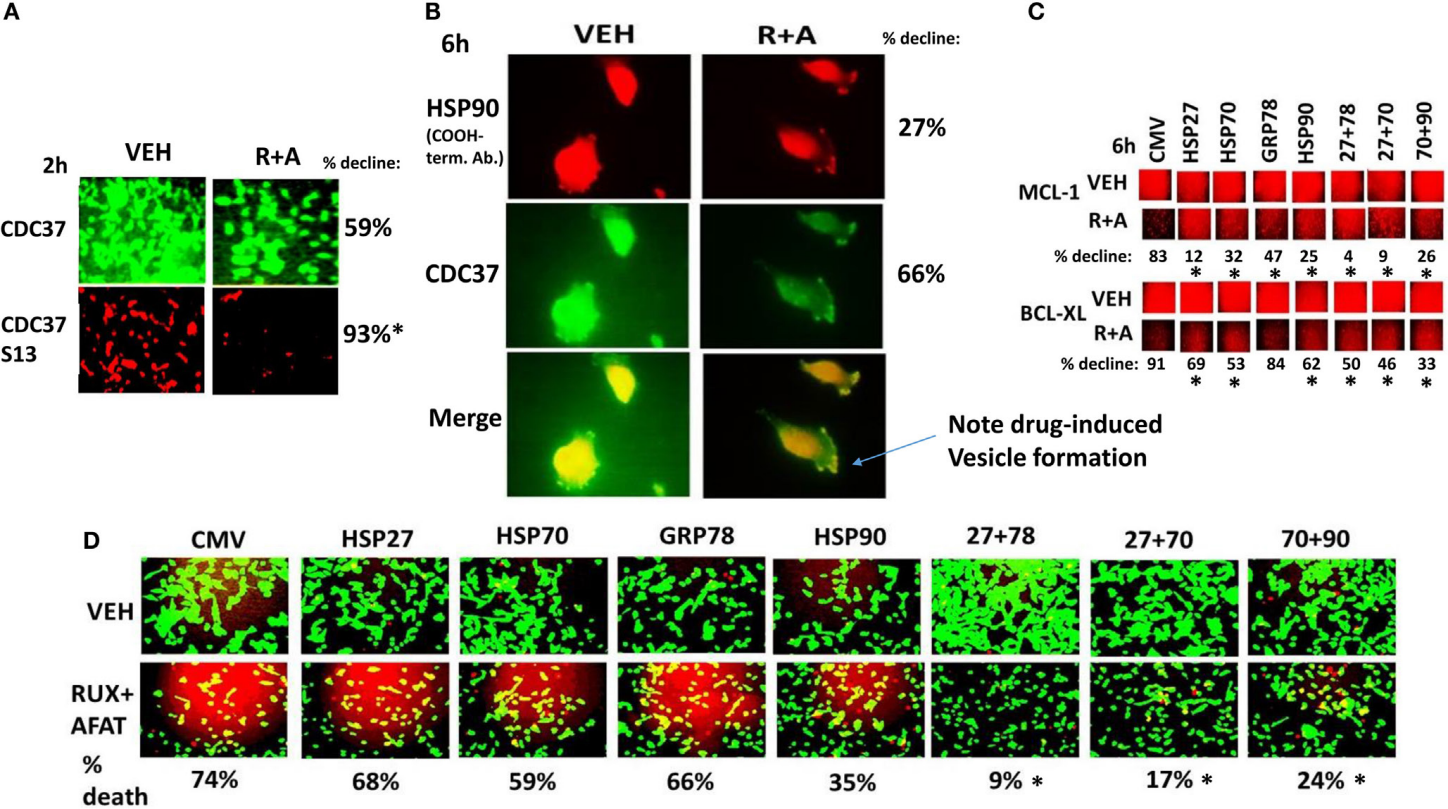
**(Ruxolitinib + afatinib) rapidly reduces CDC37 Serine 13 phosphorylation and after 6 h CDC37 total expression**. **(A,B)** SUM149 cells were treated with vehicle control or with (ruxolitinib + afatinib) for 2 h or for 6 h after which cells were fixed in place and permeabilized using 0.5% Triton X100. Immuno-fluorescence was performed: **(A)** at 10× magnification to detect the total expression and phosphorylation levels of CDC37 and CDC37 Serine 13, respectively; **(B)** at 60× magnification the total expression of HSP90 and the total expression of CDC37, and the co-localization of the two proteins (*n* = 3 ± SEM) *p < 0.05 greater decline than CDC37 protein expression. **(C)** SUM149 cells were transfected with empty vector control or with plasmids to express: HSP90, GRP78, HSP70, or HSP27, alone or in the indicated combinations. Twenty-four hours after transfection, cells were treated with vehicle control or with [ruxolitinib (1.0 μM) + afatinib (1.0 μM)] for 6 h after which cells were fixed in place and permeabilized using 0.5% Triton X100. Immuno-fluorescence was performed at 10× magnification to detect the total expression of MCL-1 and BCL-XL (*n* = 3 ± SEM) *p < 0.05 lower decline than vehicle control. **(D)** SUM149 cells were transfected with empty vector control or with plasmids to express: HSP90, GRP78, HSP70, or HSP27, alone or in the indicated combinations. Twenty-four hours after transfection, cells were treated with vehicle control or with [ruxolitinib (1.0 μM) + afatinib (1.0 μM)] for 24 h. Twenty-four hours later, cell viability was assessed using a live/dead assay in a Hermes WiScan microscope at 10× magnification (*n* = 3 ± SEM) *p < 0.05 greater survival than individual expression of chaperones.

Chaperone proteins regulate the survival of cells through multiple mechanisms, and chaperone proteins are often over-expressed in tumor tissue when compared to matched-normal tissues, thus arguing for a therapeutic window between normal and tumor tissues for use of chaperone inhibitors (30–32). In the recent Booth et al. manuscript examining multi-kinase inhibitors as chaperone inhibitors, we demonstrated that the multi-kinase inhibitors sorafenib and pazopanib are potent HSP90 and HSP70 ATPase inhibitors, but that neither afatinib nor ruxolitinib are direct chaperone ATPase inhibitors (30). We discovered that in addition to reducing total HSP90 expression, (ruxolitinib + afatinib) treatment also rapidly reduced the phosphorylation of the HSP90 essential co-chaperone CDC37 at Serine 13 within 2 h (Figure 8A). (Ruxolitinib + afatinib) treatment reduced total CDC37 expression over a longer 6-h time course as well as modestly reducing the co-localization of the chaperone HSP90 with CDC37 (Figure 8B; the co-localization image has changed from pure yellow with vehicle to red-orange after (ruxolitinib + afatinib) exposure). Over-expression of GRP78, HSP70, or HSP90 maintained to varying extents the expression of the cyto-protective MCL-1 and BCL-XL proteins during (ruxolitinib + afatinib) exposure (Figure 8C, *p* < 0.05). Over-expression of (HSP27 + GRP78), (HSP27 + HSP70), or (HSP70 + HSP90) all protected cells from (ruxolitinib + afatinib) lethality (Figure 8D). Over-expression of (HSP70 + HSP90) and of GRP78 prevented the dephosphorylation of mTOR and the increased phosphorylation of eIF2α, respectively (data not shown). Thus, inactivation of ERK1/2, AKT, mTOR, and STAT3 also facilitates cell killing by reducing the expression of BCL-XL/MCL-1 and well as through the decreased protein expression of chaperone proteins that play diverse cyto-protective roles.

**Figure 9:**
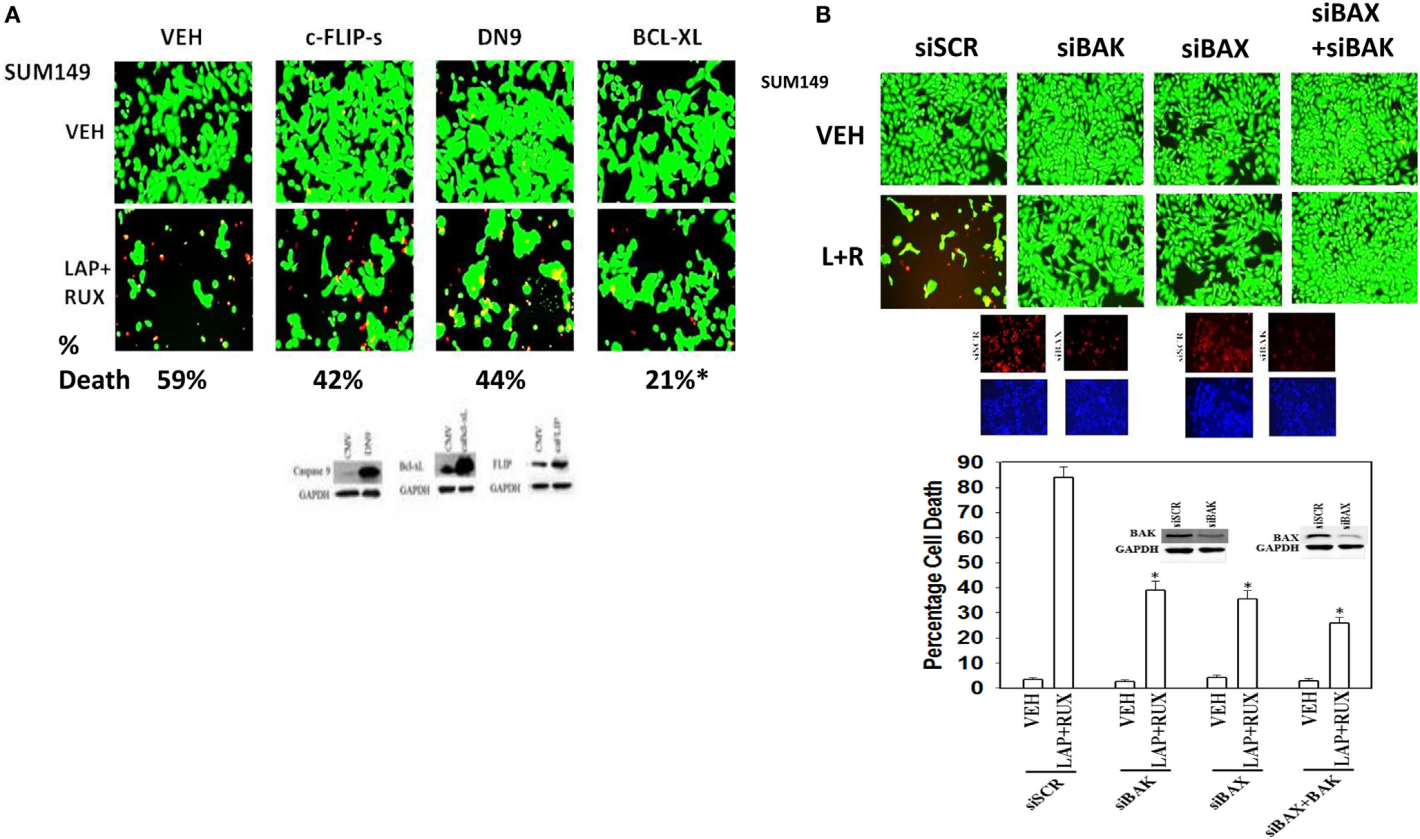
**Lapatinib and Ruxolitinib combine to kill tumor cells through apoptotic, necroptotic, and autophagic pathways (I)**. **(A)** SUM149 cells were transfected with an empty vector control plasmid (CMV) or plasmids to express: c-FLIP-s; dominant negative caspase 9 or BCL-XL. Twenty-four hours after transfection, cells were treated with vehicle control, ruxolitinib (1 μM) and/or lapatinib (1.0 μM). Twenty-four hours later, cell viability was assessed using a live/dead assay in a Hermes WiScan microscope at 10× magnification. **(B)** SUM149 cells were transfected with a scrambled siRNA (siSCR) or siRNA molecules to knock down the expression of BAK, BAX, or BAK and BAX together. Twenty-four hours after transfection, cells were treated with vehicle control, ruxolitinib (1 μM) and/or lapatinib (1.0 μM). Twenty-four hours later, cell viability was assessed using a live/dead assay in a Hermes WiScan microscope at 10× magnification. *Lower*: graphical representation of the data (*n* = 3 ± SEM) *p < 0.05 less than corresponding value in siSCR cells.

Based on our multiplex antibody array data as well as our immuno-fluorescence data; caspase 3 was being cleaved (activated) after drug combination treatment, and we next investigated the molecular mechanisms by which (ruxolitinib + ERBB1/2/4 inhibitor) was killing tumor cells. Initial studies using pharmacologic tools demonstrated that killing induced by the (ruxolitinib + lapatinib) drug combination was blocked/reduced by inhibition of: RIP-1 (necroptosis; necrostatin 1); Vps34 (autophagy; 3-methyl adenine); and caspases (apoptosis; zVAD) (data not shown). Inhibition of caspase 8/death receptor signaling by over-expression of c-FLIP-s or inhibition of caspase signaling downstream of mitochondria by expression of dominant negative caspase 9 *did not* significantly reduce the lethality of (ruxolitinib + lapatinib) treatment (Figure 9A) (34, 35). This is of note because although the drug combination causes pro-caspase 3 cleavage, the actions of this apoptotic enzyme are not required to execute the cancer cell. Over-expression of BCL-XL or knock down of either BAX or BAK strongly reduced drug combination killing (Figures 9A,B). Although knock down of PUMA or NOXA was protective against the drug combination, the effect of either individual knock down on maintaining cell viability was less than that of inhibiting both BAX and BAK function together.

**Figure 10:**
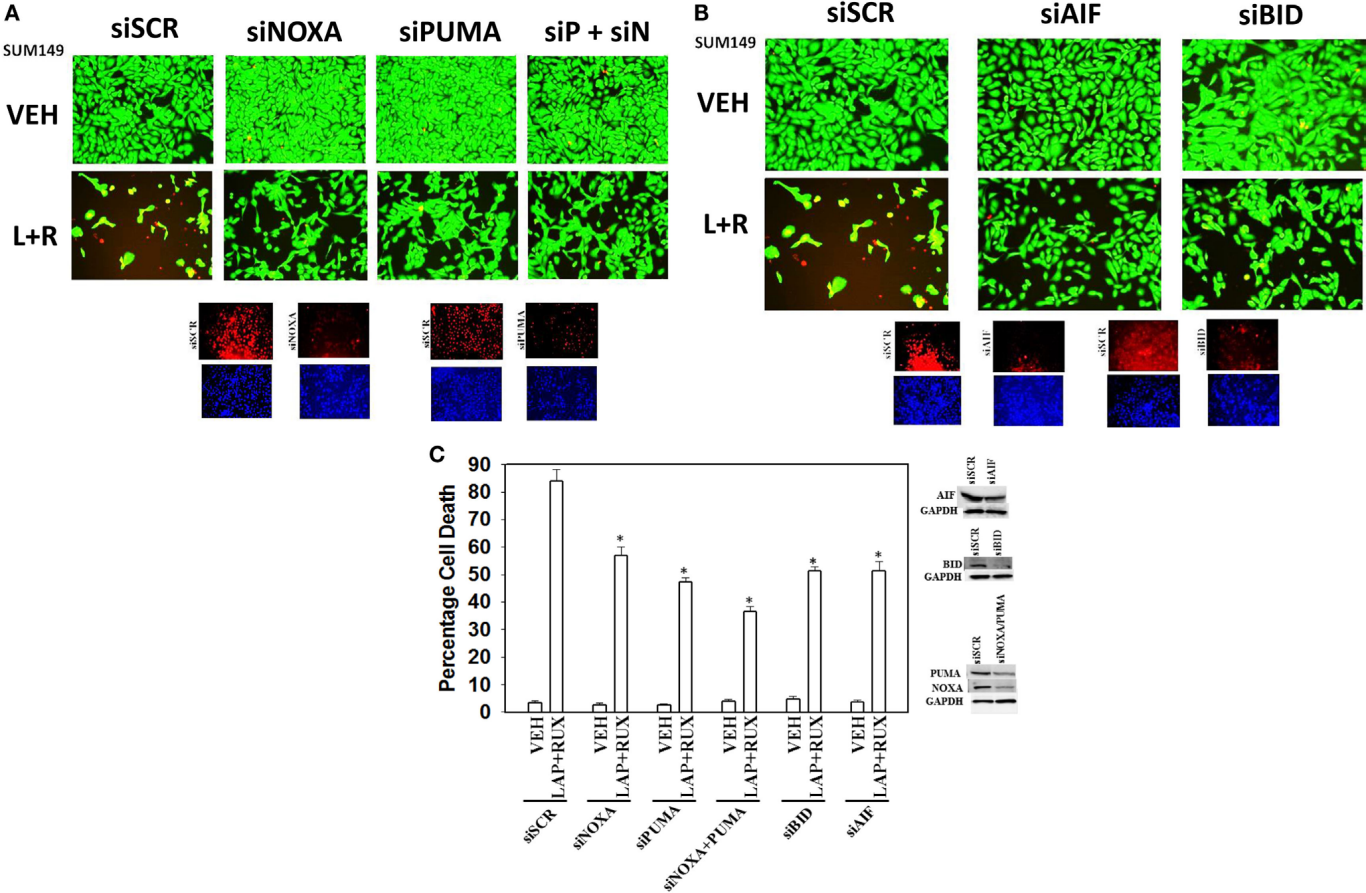
**Lapatinib and Ruxolitinib combine to kill tumor cells through apoptotic, necroptotic, and autophagic pathways (II)**. **(A)** SUM149 cells were transfected with a scrambled siRNA (siSCR) or siRNA molecules to knock down the expression of NOXA, PUMA, or NOXA and PUMA together. Twenty-four hours after transfection, cells were treated with vehicle control, ruxolitinib (1 μM) and/or lapatinib (1.0 μM). Twenty-four hours later, cell viability was assessed using a live/dead assay in a Hermes WiScan microscope at 10× magnification. **(B)** SUM149 cells were transfected with a scrambled siRNA (siSCR) or siRNA molecules to knock down the expression of BID or of apoptosis-inducing factor (AIF). Twenty-four hours after transfection, cells were treated with vehicle control, ruxolitinib (1 μM) and/or lapatinib (1.0 μM). Twenty-four hours later, cell viability was assessed using a live/dead assay in a Hermes WiScan microscope at 10× magnification. **(C)** Graphical representation of the data (*n* = 3 ± SEM) *p < 0.05 less than corresponding value in siSCR cells.

The toxic BH3 domain protein BID is activated by proteolytic cleavage; a cleavage most commonly thought to be catalyzed by caspases 8 and 10 downstream of death receptors but also less commonly by cathepsin and calpain proteases released due to lysosomal dysfunction/autophagy flux (27). While over-expression of c-FLIP-s did not prevent the (ruxolitinib + lapatinib) drug combination from killing, i.e., there is no caspase 8 signaling component being induced; knock down of BID very clearly did significantly reduce cell killing (Figures 10A,B, *p* < 0.05). In agreement with there being a caspase-independent mechanism for drug combination killing also *downstream of the mitochondrion*, knock down of apoptosis-inducing factor (AIF) expression reduced cell death; *n.b*. and, as we demonstrated in a prior data set, dominant negative caspase 9 was not protective (Figures 10B,C).

**Figure 11:**
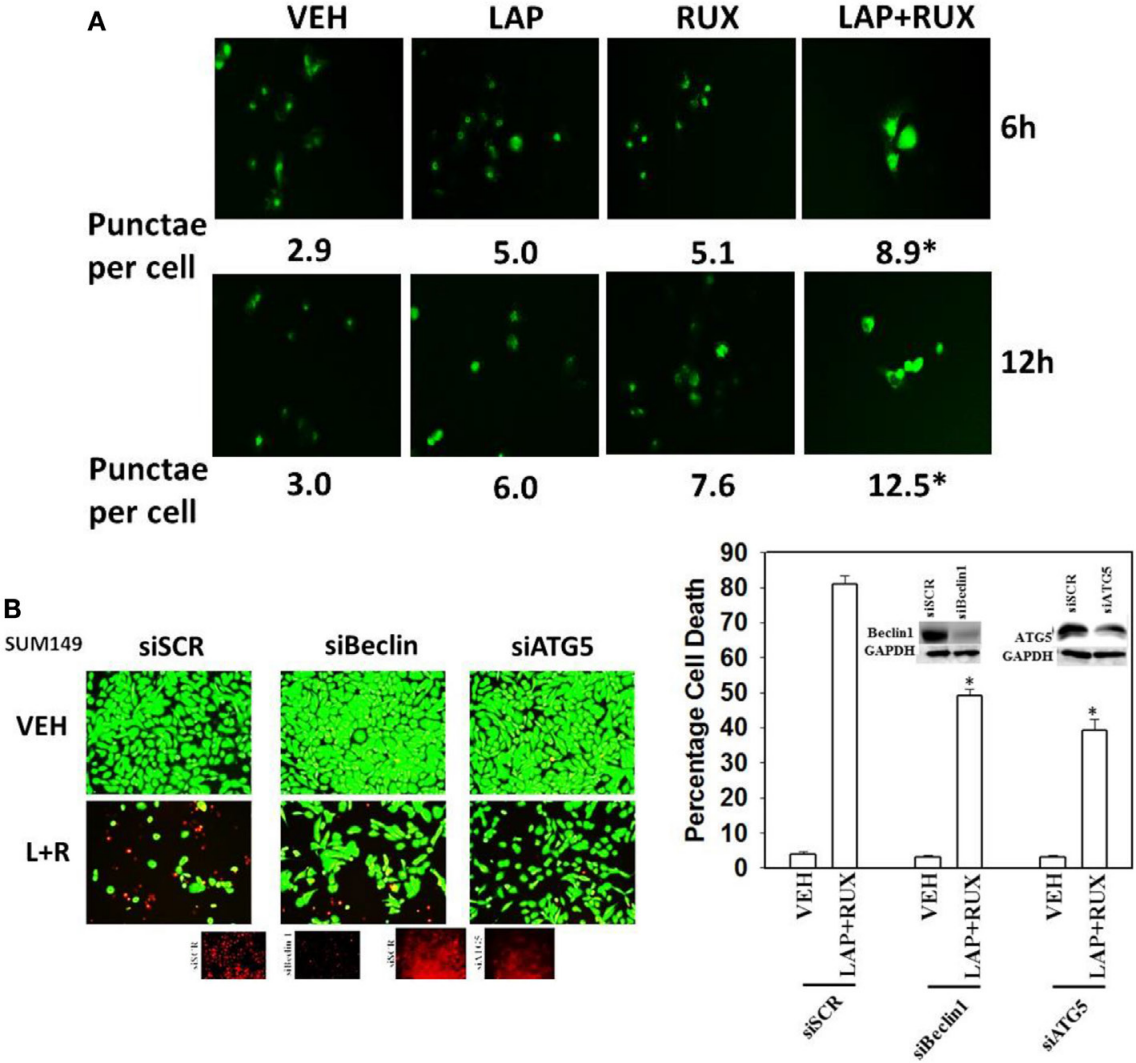
**Lapatinib and Ruxolitinib interact to stimulate autophagosome formation**. **(A)** SUM149 cells were transfected with a plasmid to express LC3-GFP. Twenty-four hours after transfection, cells were treated with vehicle control, lapatinib (1 μM), ruxolitinib (1 μM), or in combination as indicated for 6 h or for 12 h. At each time point, cells were visualized using a fluorescent microscope at 40× magnification, and the mean number of intense punctate GFP-positive vesicles per cell determined in >40 cells from random fields ± SEM (*p < 0.05 greater than lapatinib alone or ruxolitinib alone treatments). **(B)** SUM149 cells were transfected with a scrambled siRNA (siSCR) or siRNA molecules to knock down the expression of Beclin1 or of ATG5. Twenty-four hours after transfection, cells were treated with vehicle control, ruxolitinib (1 μM), and/or lapatinib (1.0 μM). Twenty-four hours later, cell viability was assessed using a live/dead assay in a Hermes WiScan microscope at 10× magnification (*n* = 3 ± SEM) *p < 0.05 less than corresponding value in siSCR cells.

**Figure 12:**
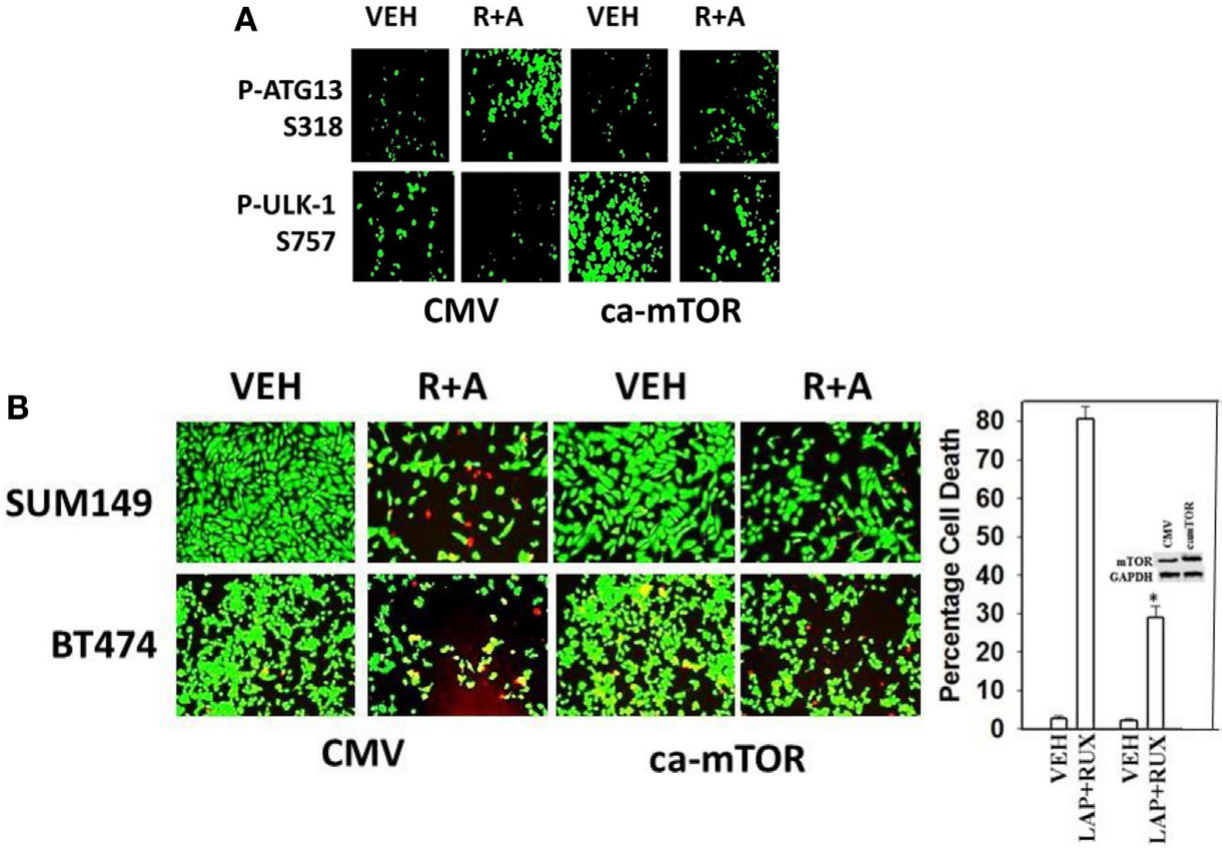
**Lapatinib/Afatinib and Ruxolitinib kill tumor cells through toxic mitophagy**. **(A)** BT474 cells were transfected with empty vector plasmid (CMV) or a plasmid coding for activated mTOR. Twenty-four hours after transfection, cells were treated with vehicle control or with [ruxolitinib (1 μM) + afatinib (1 μM)] for 6 h after which cells were fixed in place and permeabilized using 0.5% Triton X100. Immuno-fluorescence was performed to detect the total expression and phosphorylation levels of the indicated proteins. **(B)** SUM149 and BT474 cells were transfected with empty vector plasmid (CMV) or a plasmid coding for activated mTOR. Twenty-four hours after transfection, cells were treated with vehicle control, ruxolitinib (1 μM) and afatinib (1.0 μM). Twenty-four hours later, cell viability was assessed using a live/dead assay in a Hermes WiScan microscope at 10× magnification (*n* = 3 ± SEM) *p < 0.05 less than corresponding value in CMV cells.

As non-caspase dependent activation of BID was involved in cell killing for the drug combination, and as the drug combination enhanced Beclin1 expression, and as the proteases usually responsible for this biology are stored in the lysosomes, we explored whether altered levels of autophagy played any role in (ruxolitinib + lapatinib) toxicity. Treatment of cells with (ruxolitinib + lapatinib) increased the numbers of autophagosomes in cells in a greater-than-additive fashion and in a time-dependent fashion (Figure 11A, *p* < 0.05). In agreement with our preliminary data using 3-methyl adenine, and our ER stress data showing increased Beclin1 expression, knock down of Beclin1 or of ATG5 suppressed, but did not completely abolish, tumor cell killing by the (ruxolitinib + lapatinib) drug combination (Figure 11B). In agreement with our data showing mTOR dephosphorylation at S2448 and S2481, treatment of cells with (ruxolitinib + afatinib) decreased the phosphorylation of ULK-1 S757 by >50%, resulting in ULK-1 activation as shown by elevated ATG13 S318 phosphorylation by >50% which correlated with autophagosome formation (Figure 12A, *p* < 0.05). Expression of an activated form of mTOR prevented drug-induced phosphorylation of ATG13 S318, prevented autophagosome formation, and significantly reduced tumor cell killing (Figure 12B, data not shown).

**Figure 13:**
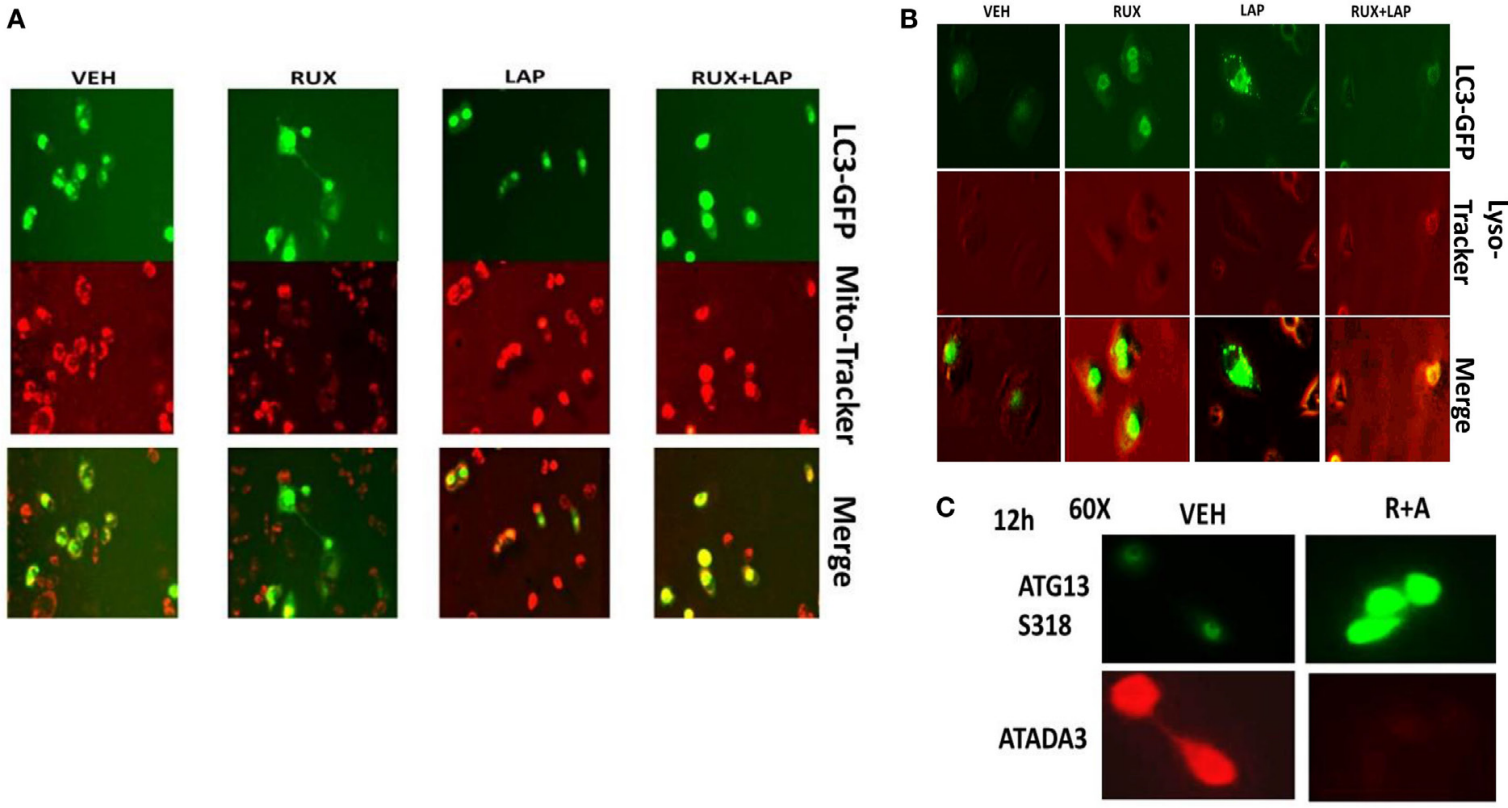
**Ruxolitinib and lapatinib interact to stimulate mitophagy**. **(A)** SUM149 cells were transfected with a plasmid to express LC3-GFP. Twenty-four hours after transfection, cells were treated with vehicle control, lapatinib (1 μM), ruxolitinib (1 μM), or in combination as indicated for 6 h. Cells were then treated with mito-tracker red (100 nM) for 15 min. Cells were rapidly visualized using a fluorescent microscope at 40× magnification in the red and green fluorescent channels. The red and green images were merged in Adobe Photoshop CS6; areas of yellow staining indicate the co-localization of GFP and mito-tracker red staining. **(B)** SUM149 cells were transfected with a plasmid to express LC3-GFP. Twenty-four hours after transfection, cells were treated with vehicle control, lapatinib (1 μM), ruxolitinib (1 μM), or in combination as indicated for 12 h. Cells were then treated with lyso-tracker red (100 nM) for 15 min. Cells were rapidly visualized using a fluorescent microscope at 40× magnification in the red and green fluorescent channels. The red and green images were merged in Adobe Photoshop CS6; areas of yellow staining indicate the co-localization of GFP and lyso-tracker red staining. **(C)** SUM149 cells were treated with vehicle control or with [ruxolitinib (1 μM) + afatinib (0.5 μM)] for 12 h, after which cells were fixed in place and permeabilized using 0.5% Triton X100. Immuno-fluorescence was performed to detect the expression of ATAD3A and ATG13 S318 with images at 60× magnification.

**Figure 14:**
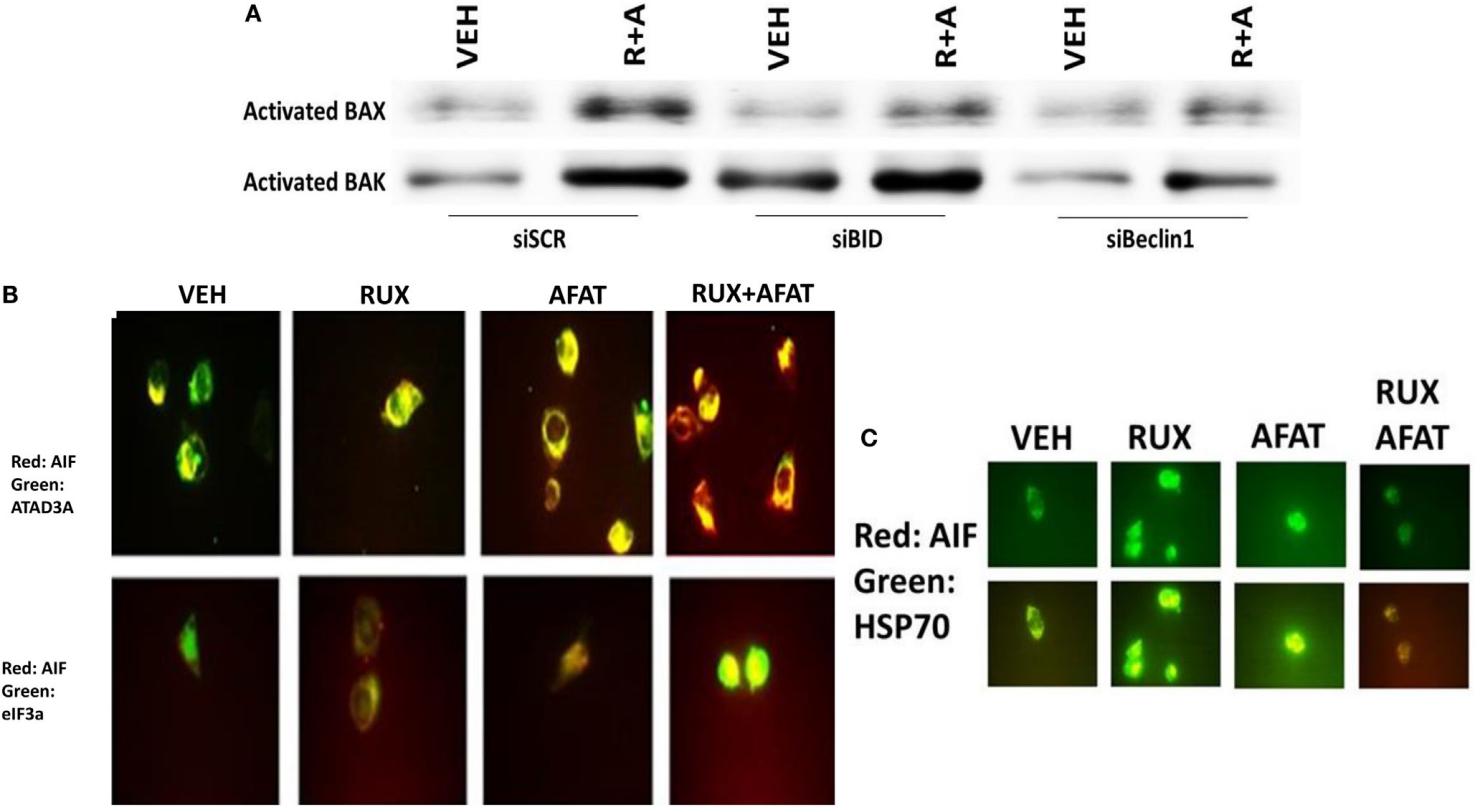
**Activation of BAX and BAK requires the initial induction of autophagy**. **(A)** SUM149 cells were transfected with a scrambled nonsense siRNA molecule (SCR), or siRNA molecules to knock down the expression of BID or of Beclin1. Twenty-four hours after transfection, cells were treated for 6 h with vehicle control (VEH) or with [ruxolitinib (1.0 μM) + afatinib (1.0 μM) for 6 h. Immuno-precipitates of active BAX and of active BAK are run on SDS-PAGE (14%) and electrophoresed proteins transferred to 0.22-μm-thick nitrocellulose membranes for immunoblotting against the total expression of BAX and of BAK under each condition. **(B)** SUM149 cells were treated with vehicle control or with [ruxolitinib (1 μM) and/or afatinib (1.0 μM)] as indicated for 6 h after which cells were fixed in place and permeabilized using 0.5% Triton X100. Immuno-fluorescence was performed at 60× magnification to detect the co-localization levels of the indicated proteins, and for the total expression level of AIF. **(C)** SUM149 cells were treated with vehicle control or with [ruxolitinib (1 μM) and/or afatinib (1.0 μM)] for 6 h after which cells were fixed in place and permeabilized using 0.5% Triton X100. Immuno-fluorescence was performed at 60× magnification to detect the co-localization levels of the indicated proteins, and the expression of HSP70 using a COOH terminal epitope antibody.

In agreement with our ATG13 S318 phosphorylation and activated mTOR viability data, treatment of cells with (ruxolitinib + lapatinib) caused LC3-GFP intense staining vesicles to co-localize with mitochondria (Figure 13A). Treatment of cells with (ruxolitinib + lapatinib) also caused LC3-GFP staining vesicles to co-localize with acidic lysosomes (Figure 13B). That is to say, we are causing mitophagy and autophagic flux. In agreement with the induction of mitophagy, the expression of the mitochondrial protein ATADA3 declined 12 h after exposure in parallel to increased ATG13 S318 phosphorylation (Figure 13C). We next determined whether the induction of autophagosome formation was an essential process for activation of BAX and BAK above the level of mitochondrial dysfunction or whether it was a consequence downstream of an early form of mitochondrial dysfunction. Treatment of SUM149 cells with (ruxolitinib + afatinib) activated within 6 h the toxic BH3 domain proteins BAX and BAK (Figure 14A). Activation of BAX was almost abolished by knock down of Beclin1 or of BID expression, whereas activation of BAK was only modestly impacted by BID knock down.

Apoptosis-inducing factor is a mitochondrial protein that is released into the cytoplasm upon a lethal stimulus, and it then re-locates to the nucleus where it acts to promote a non-caspase dependent fragmentation of the DNA. In unstimulated BT474 cells, AIF co-localized with the mitochondrial protein ATAD3A but not with the nuclear protein eIF3A (Figure 14B). Six hours after treatment with (ruxolitinib + afatinib), the co-localization of AIF with eIF3A in the nucleus had increased and notably that ATAD3A levels did not alter suggesting that complete destructive mitophagy had not occurred at this time point. The chaperone HSP70 can bind to the released AIF protein in the cytosol where it inhibits AIF translocation to the nucleus, thereby blocking AIF-dependent killing; our prior data in this paper had shown chaperone expression, including that of HSP70, was being reduced by (ruxolitinib + afatinib). Treatment of cells with (ruxolitinib + afatinib) resulted in reduced expression of HSP70 and reduced co-localization of AIF with HSP70 in the cytosol (Figure 14C). That is to say, both the ruxolitinib alone and afatinib alone treatments caused a translocation of AIF into the cytosol where it associated with HSP70 at similar levels based on the “pure” yellow fluorescent stain, whereas in cells treated with (ruxolitinib + afatinib) the cellular fluorescent stain was a “dirty” reddish-yellow. Collectively, these protein co-localization data suggest that under these treatment conditions, there was cytosolic red fluorescent AIF protein that was not associated with cytosolic green fluorescent HSP70.

**Figure 15:**
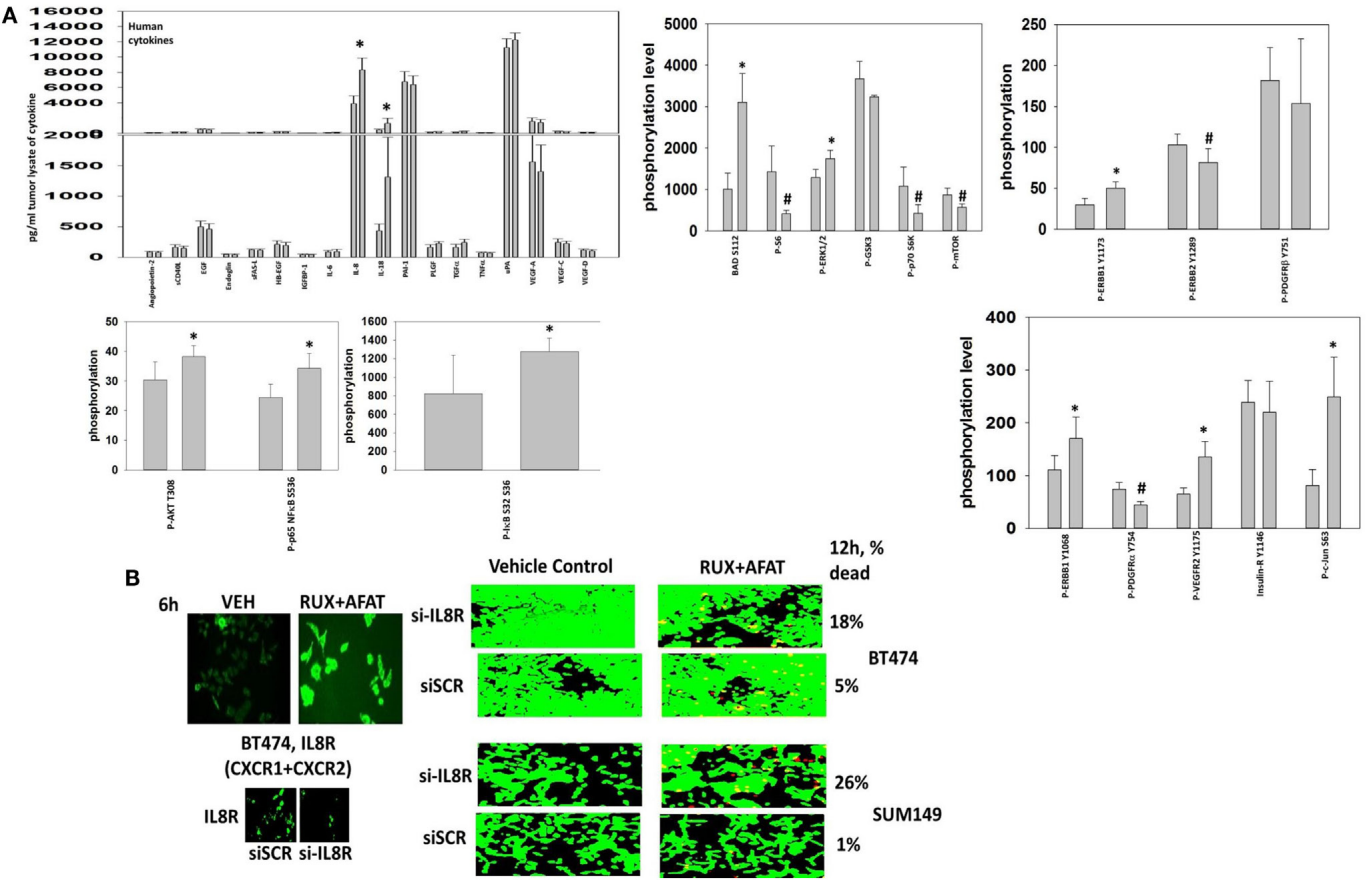
**Multiplex antibody array assays demonstrate that tumors previously exposed to (ruxolitinib + afatinib) exhibit elevated expression of IL-8 and IL-18**. **(A)** Tumor material, isolated at day 35, was homogenized and prepared for the multiplex assays as under the manufacturer’s instructions. Multiplex assays were performed in a Bio-Rad MAGPIX machine using the multiplex plates as discussed in the section “Methods.” Data are presented as the mean of five separate control and (ruxolitinib + afatinib) tumors (± SD). *p < 0.05 greater than control value; #*p* < 0.05 less than control value. **(B)** left. BT474 tumor cells *in vitro* were treated with vehicle control or [ruxolitinib (2 μM) + afatinib (2 μM)] for 6 h. After 6 h cells were fixed in place without being permeabilized. Immuno-fluorescence was performed to detect the cell surface expression of the IL-8 receptor (right). BT474 cells and SUM149 cells were transfected with a scrambled siRNA or a siRNA to knock down expression of the IL-8 receptor. Twenty-four hours after transfection, cells are treated with vehicle control or [ruxolitinib (2 μM) + afatinib (2 μM)] for 24 h. Cell viability was assessed using a live/dead assay in a Hermes WiScan microscope at 10× magnification.

Finally, with the intention to initiate in the near future a phase I dose-limiting toxicity trial combining (ruxolitinib + an ERBB1/2/4 inhibitor) in patients at Massey Cancer Center, we performed additional multiplex antibody array analyses on tumor material from the (ruxolitinib + afatinib) BT474 tumor growth experiment to identify possible response biomarkers in tumor cells that had survived and re-grown after drug treatment. Control-treated BT474 tumors and tumors that had survived and re-grown after (ruxolitinib + afatinib) therapy were homogenized and processed for multiplex assays. The re-grown (ruxolitinib + afatinib) exposed tumors exhibited significantly higher expression levels of the cytokines *human* IL-8 and *human* IL-18 (Figure 15A). Similar data were observed for *human* CXCL-1 (not shown). In other words, (ruxolitinib + ERBB1/2/4 inhibitor) treatment initially reduces CXCL-1, IL-8, and IL-18 expression that then rebounds in the surviving tumor cells, being over-expressed (Figure 15A) *cf in vitro* multiplex data in (Figure 3). Re-grown mammary tumors that were exposed to (ruxolitinib + afatinib) also exhibited significantly higher levels of phosphorylated p65 NFκB S536, IκB S32 S36, c-Jun S63, AKT T308, ERBB1 Y1173/Y1068, VEGFR2 Y1175, BAD S112, and ERK1/2, and significantly lower levels of ERBB2 Y1289, p70 S6K T389 and mTOR S2448 and P-S6 phosphorylation. The phosphorylation changes in NFκB/IκB together argue for activation of NFκB transcriptional signaling in the re-grown tumor cells. (Ruxolitinib + afatinib)-treated tumors, in agreement with lower levels of mTOR activity, had higher basal ATG13 S318 phosphorylation and an elevated basal level of autophagosomes (data not shown).

Treatment of BT474 tumor cells *in vitro* with (ruxolitinib + afatinib) increased the expression of the IL-8 receptors CXCR1 and CXCR2 on the cell surface (Figure 15B). Combined molecular knock down of both of the IL-8 receptors CXCR1 and CXCR2 modestly though significantly enhanced the lethality of (ruxolitinib + afatinib) treatment in both BT474 and SUM149 cells (*p* < 0.05). This suggests that CXCR1 and CXCR2 signaling provides the surviving re-grown tumor cells with a paracrine loop from IL-8 and CXCL-1 to maintain tumor cell survival. Whether this also alters immune cell infiltration into the tumor and, thus, tumor sensitivity to checkpoint inhibitors will require detailed additional studies.

Collectively, our data argue that after (ruxolitinib + afatinib) drug combination exposure, an ERBB1-(ERK + AKT)-NFκB pathway becomes activated in surviving BT474 cells to promote “recurrent” tumor growth; as BAD S112 phosphorylation is increased, this pathway also likely acts to reduce the apoptotic threshold by inactivating BAD. Studies beyond the scope of the present manuscript and our data with IL-8 receptor and IL-18 receptor signaling will be required to prove whether this signaling “fingerprint” in the re-grown tumors can be used as a template for designing new anti-tumor drug combinations.

## Discussion

The present studies were undertaken to determine whether the myelo-proliferative disorder medication ruxolitinib (Jakafi^®^) could be repurposed as a solid tumor cancer therapeutic. We discovered that ruxolitinib at clinically relevant free drug concentrations synergized with multiple ERBB1/2/4 inhibitors to kill tumor cells, including those expressing mutated active RAS proteins or lacking the tumor suppressor PTEN. Unlike the drugs OSU-03012 (AR12), sorafenib or pazopanib, neither afatinib nor ruxolitinib inhibited the ATPase activities of cyto-protective chaperone proteins but instead, in combination, reduced the *protein expression* of the HSP90 and HSP70 chaperones (30–32). That the protein expression of both HSP90 and HSP70 were reduced argues that our drug combination may utilize multiple pathways in any given tumor cell to cause cell death, explaining why cells that expressed activated oncogenes, such as RAS, or had inactivated tumor suppressor genes, such as PTEN, were all drug-combination sensitive.

Tumor cell killing by (ruxolitinib + ERBB inhibitor) treatment occurred in a wide variety of tumor cell types, including many genetically diverse PDX models of GBM and lung cancer. Multiple first-, second-, and third-generation ERBB1/2/4 inhibitors could interact with ruxolitinib to kill tumor cells. Knock down of ERBB1/2/3/4 protein expression in the three tumor cell isolates we examined recapitulated the toxic interaction of the ERBB1/2/4 inhibitors with ruxolitinib. Similarly, we noted that combined knock down of JAK1 and JAK2 could recapitulate the toxic effects of ruxolitinib in a cell type-dependent fashion when combined with ERBB1/2/4 inhibitors. There is another Janus kinase inhibitor that is approved by the FDA, for the treatment of rheumatoid arthritis, tofacitinib (Xeljanz^®^). Tofacitinib is a JAK3 inhibitor and demonstrates some JAK1 pathway inhibitory effects in mouse models of arthritis. In contrast to ruxolitinib, tofacitinib did not consistently enhance the lethality of lapatinib or of MMF in our four PDX GBM isolates [(33, Tavallai and Dent, Unpublished Observations^1^)]. Tofacitinib enhanced lapatinib toxicity and MMF toxicity only in GBM6 and GBM14 cells, but not in GBM5 or GBM12 cells. To Dent laboratory investigators such findings are conceptually important because as we experienced with our in depth analyses of OSU-03012 (AR12) biology, originally proposed to be an inhibitor of PDK-1 in the PI3K pathway, only by rational unbiased experimental studies did we eventually determine that the OSU-03012 drug is in fact a potent inhibitor of multiple cyto-protective chaperone proteins, including GRP78, HSP90, and HSP70 (30).

In both BT474 and in SUM149 mammary carcinoma cells, expression of constitutively active STAT3; or of activated AKT; or of activated MEK1 were shown to be a key individual protective molecular signals to suppress (ruxolitinib + ERBB inhibitor) – induced killing, though of additional note molecular inhibition of JNK pathway signaling could also reduce the death response. In part, activated STAT3/activated AKT/activated MEK as individual signals could partially protect tumor cells because each activated protein maintained to varying degrees: (a) the expression of the mitochondrial and endoplasmic reticulum protective proteins BCL-XL and MCL-1; (b) suppressed activation of the endoplasmic reticulum stress eIF2α-Beclin1 pathway; (c) and stabilized the levels of cyto-protective chaperone proteins, proteins whose essential pleiotropic cyto-protective effects on many signaling and cell death pathway targets will collectively collaborate to prevent drug-induced tumor cell killing (30–32).

The killing mechanisms induced by the (ruxolitinib + ERBB inhibitor) treatment were many-fold, most notably that (ruxolitinib + ERBB inhibitor) treatment caused a significant greater-than-additive increase in the levels of autophagosome formation that was associated with mitochondrial degradation and activation of caspase 3; as well as autolysosome formation, mitochondrial degradation, AIF translocation to the nucleus, and a necroptotic form of tumor cell death. Our studies strongly argued that the rapid inactivation of the PI3K–mTOR pathway caused by direct kinase inhibition and by subsequent reduced chaperone protein expression in parallel with increased PERK-eIF2α ER stress signaling leading to elevated Beclin1 levels were the key upstream pathways by which tumor cell death was being triggered. The confluence of these signaling events was the increase in autophagosome formation; autophagosomes that co-localized with mitochondria (mitophagy) and then with lysosomes (autolysosomes). Activation of the executioner toxic BH3 domain proteins BAX and BAK required increased Beclin1 expression (autophagosome formation) and also the activation/cleavage of BID due to the release into the cytosol of cathepsins and calpains (autolysosome disintegration).

One chaperone-associated protein that plays a key survival regulatory role downstream of the mitochondrion, and that we validated in our drug-combination system as a probable chaperone effector for cell killing, as has been observed by others, is AIF. The chaperone HSP70, often over-expressed in tumors, can sequester and inactivate cytosolic AIF previously released from the mitochondrion, thereby preventing AIF translocating to the nucleus where it would trigger tumor cell death. That our drug combination: (a) reduced HSP70: AIF co-localization in the cytosol; (b) increased eIF3A: AIF association in the nucleus; and (c) reduced total HSP70 expression, strongly argues that the upstream mTOR inhibition/ER stress-autophagy-BAX/BAK/BID-dependent AIF release from the mitochondria and the facilitation of its translocation to the nucleus is very likely the key mechanism by which our (ruxolitinib + afatinib) drug combination was killing tumor cells.

One additional commonality in our studies exploring the molecular mechanisms by which (ruxolitinib + ERBB inhibitors) killed tumor cells, based on our siRNA analyses of ERBB family receptors, was the importance of signaling through ERBB3, a receptor lacking tyrosine kinase activity but containing six sites of tyrosine phosphorylation that can become acceptor sites for the p85 SH2 domain of PI3K (36, 37). ERBB3 is phosphorylated by ERBB1/2/4 and also by SRC family kinases. Downstream of ERBB3 lies the PI3K/AKT/mTOR pathway, and constitutive AKT signaling or mTOR signaling was protective against (ruxolitinib + ERBB inhibitor) exposure. Activated AKT expression could maintain BCL-XL and MCL-1 levels during drug exposure, as well as maintain the inactivating phosphorylation of BAD S112/S136. By reducing BAD phosphorylation thereby increasing BAD activity, the drug combination would be predicted to facilitate killing by cleaved BID and activated forms of BAX and BAK. We found via multiplex assays that the pro-apoptotic protein BAD was hyper-phosphorylated in tumors previously exposed to (ruxolitinib + afatinib), and is phosphorylated on Serine 112 by ERK1/2; in preliminary studies the drug combination reduced BAD S112 phosphorylation in these tumor cells by ~50%; knock down of BAD reduced the lethality of (ruxolitinib + afatinib) and also reduced drug-induced activation of BAX [Observations^1^].

From our multiplex assays on tumor material that had re-grown after drug combination exposure, we discovered that the basal expression levels of multiple cyto-protective growth factors and cytokines had been elevated. In a paracrine fashion, these factors, such as IL-8 and CXCL-1, as a collective will help to maintain AKT and mTOR activity in the face of drug combination exposure, and also in the face of reduced HSP90/HSP70 expression that acts to maintain signaling through the PI3K–mTOR pathway. Hence, we conclude that a simplistic one-size-fits-all detailed mechanism by which (ruxolitinib + ERBB inhibitor) exposure kills all tumor cell isolates all of the time cannot be categorically *a priori* defined.

In conclusion, (ruxolitinib + ERBB1/2/4 inhibitors) treatment kills genetically diverse tumor cells by facilitating autophagosome and autolysosome formation over the course of 6–12 h through inactivation of HSP90/HSP70-mTOR and activation of eIF2α-Beclin1 pathways. Autophagy-induced activation of BAX and BAK stimulates the release of AIF into the cytosol with subsequent mitochondrial break-down in autolysosomes. The cytosolic AIF that, due to reduced HSP70 function/expression, can more freely translocate to the nucleus where it then causes DNA degradation and tumor cell death. Studies beyond the scope of the present paper will be required to more completely understand how (ruxolitinib + afatinib) interact to regulate autophagy to kill and whether (ruxolitinib + afatinib) is a drug combination that we can translate into the clinic for solid tumor patients.

## Author Contributions

PD, MT, and AP participated in research design; PD and MT originally conceived of the (ruxolitinib + ERBB inhibitor) drug combination concept; PD conceptualized the use of MMF, and during performance of the studies AP provided ideas for animal studies and multiplex experiments that would provide biomarkers for clinical translation trials. MT and LB performed *in vitro* cell biology assays using the Hermes system with both authors each performing the initial or repeat studies. MT performed the western immunoblotting studies. JR and MT performed the animal studies and also determined tumor volumes. JR performed the multiplex assays. Statistical analyses of experimental data were performed using Sigma-Plot software by PD, LB, and MT. PD and MT drafted the manuscript that is in part the PhD dissertation of MT. All authors read and approved the final manuscript.

## Conflict of Interest Statement

The authors declare that the research was conducted in the absence of any commercial or financial relationships that could be construed as a potential conflict of interest.

## Footnotes

^1^In preliminary studies we discovered that the drug combination reduced BAD S112 phosphorylation by ~50% and that knock down of BAD reduced the lethality of (ruxolitinib + afatinib) and also reduced drug-induced activation of the toxic BH3 domain protein BAX. Methods used were identical to those in published Figures in the manuscript.

## Abbreviations

ca, constitutively active; cCasp3, cleaved caspase 3; CMV, empty vector plasmid or virus; dn, dominant negative; ER, endoplasmic reticulum; ERK, extracellular regulated kinase; IP, immunoprecipitation; JAK, Janus Kinase; MAPK, mitogen-activated protein kinase; MEK, mitogen-activated extracellular regulated kinase; mTOR, mammalian target of rapamycin; PI3K, phosphatidyl inositol 3 kinase; PTEN, phosphatase and tensin homolog on chromosome ten; ROS, reactive oxygen species; SCR, scrambled; si, small interfering; STAT, signal transducers and activators of transcription; VEH, vehicle.
